# Olympic Sports Science—Bibliometric Analysis of All Summer and Winter Olympic Sports Research

**DOI:** 10.3389/fspor.2021.772140

**Published:** 2021-10-20

**Authors:** Grégoire P. Millet, Franck Brocherie, Johannes Burtscher

**Affiliations:** ^1^Institute of Sport Sciences, University of Lausanne, Lausanne, Switzerland; ^2^Department of Biomedical Sciences, University of Lausanne, Lausanne, Switzerland; ^3^Laboratory Sport Expertise and Performance (EA 7370), French Institute of Sport, Paris, France

**Keywords:** citations, publication, sport sciences, summer Olympic sports, winter Olympic sports

## Abstract

**Introduction:** The body of scientific literature on sports and exercise continues to expand. The summer and winter Olympic games will be held over a 7-month period in 2021–2022.

**Objectives:** We took this rare opportunity to quantify and analyze the main bibliometric parameters (i.e., the number of articles and citations) across all Olympic sports to weigh and compare their importance and to assess the structure of the “sport sciences” field. The present review aims to perform a bibliometric analysis of Olympic sports research. We quantified the following topics: (1) the most investigated sports; (2) the main journals in which the studies are published; (3) the main factors explaining sport-specific scientific attractiveness; (4) the influence of being in the Olympic programme, economic weight, and local influences on research output; and (5) which research topic is the most investigated across sports.

**Methods:** We searched 116 sport/exercise journals on PubMed for the 40 summer and 10 winter Olympic sports. A total of 34,038 articles were filtered for a final selection of 25,003 articles (23,334 articles on summer sports and 1,669 on winter sports) and a total of 599,820 citations.

**Results and Discussion:** Nine sports [football (soccer), cycling, athletics, swimming, distance & marathon running, basketball, baseball, tennis, and rowing] were involved in 69% of the articles and 75% of the citations. Football was the most cited sport, with 19.7 and 26.3% of the total number of articles and citations, respectively. All sports yielded some scientific output, but 11 sports (biathlon, mountain biking, archery, diving, trampoline, skateboarding, skeleton, modern pentathlon, luge, bobsleigh, and curling) accumulated a total of fewer than 50 publications. While ice hockey is the most prominently represented winter sport in the scientific literature, winter sports overall have produced minor scientific output. Further analyses show a large scientific literature on team sports, particularly American professional sports (i.e., baseball, basketball, and ice hockey) and the importance of inclusion in the Olympic programme to increasing scientific interest in “recent” sports (i.e., triathlon and rugby sevens). We also found local/cultural influence on the occurrence of a sport in a particular “sport sciences” journal. Finally, the relative distribution of six main research topics (i.e., physiology, performance, training and testing, injuries and medicine, biomechanics, and psychology) was large across sports and reflected the specific performance factors of each sport.

## Introduction

The Olympic sports (https://olympics.com/en/sports/) bring together a large and diverse range of human abilities that extend far beyond the Olympic motto, “*Citius—Altius—Fortius*” (i.e., Faster—Higher—Stronger”), and outstanding genetic, physical, technical and mental skills are required to reach an Olympic podium. It is therefore not surprising that behind each athlete is an interdisciplinary team of experts/scientists (Hodson, 2021). Elite sports performance has long been a fascinating field of research for scientists. The 1922 Nobel Prize in Physiology or Medicine, awarded to Sir A. V. Hill and his work on the best middle-distance runners of his time, provides a perfect example of ground-breaking research originating from related questions (Hill, 1925). Over the last two decades, the “sport sciences” field has massively expanded, as evidenced by the continuously growing number of journals (e.g., 85 journals in 2021 vs. 58 in 1998 in the “sport sciences” category of the Incites journal citations report—https://jcr.clarivate.com). The original definition of sport sciences as “*the study and application of scientific principles and techniques to improve sporting performance*” (Lippi et al., 2008) has become too narrow, and researchers in different scientific fields (e.g., antidoping sciences, biomechanics, physiology, nutrition, injury prevention and rehabilitation, psychology, pedagogy, management and marketing, history, sociology and many biomedical fields, including preventive medicine and oncology) (Millet and Giulianotti, 2019) are producing an enormous body of research related to exercise and sports. However, to our knowledge, there has been no comprehensive analysis of the “sport sciences” field and no comparison of the sport-specific scientific literature across all Olympic sports. Currently available bibliometric analyses are limited to the most cited articles in sport and exercise medicine (Knudson, 2011; Khatra et al., 2021) or specifically concern a single sport, such as football (soccer) (Brito et al., 2018), or a specific scientific field (e.g., sports economics, sports management or sociology) (Santos and Garcia, 2011; Shilbury, 2011; Gau, 2013).

In 1992, the summer (Barcelona) and winter (Albertville) Olympic games took place for the last time in the same year. Due to the COVID-19 pandemic, the two games (Tokyo 2020 Summer Olympic Games between 23 July and 8 August 2021 and Beijing 2022 Winter Olympics between 4 and 20 February 2022) will now be organized within a 7-month timeframe. This may be an occasion to review the science across all summer and winter Olympic sports.

The present review aims to perform a bibliometric analysis of Olympic sports research. We quantified the following topics: (1) the most investigated sports; (2) the main journals in which the studies are published; (3) the main factors explaining sport-specific scientific attractiveness; (4) the influence of being in the Olympic programme, economic weight, and local influences on research output; and (5) which research topic is the most investigated across sports.

## Methods

The data were obtained by a search in PubMed followed by a search conducted in Web of Science (Clarivate Analytics, USA). First, we selected 116 “sport sciences” journals (Table 1), including 85 journals of the “*sport sciences*” category in the Incites journal citations report (Clarivate Analytics, USA); then, we expanded the search to other journals with “exercise” or “sport” in the title. Second, we chose to limit the analysis to sports that are currently in the Olympic programme for Tokyo 2020 (Table 2A) and Beijing 2022 (Table 2B). This list of sports does not contain sports to be included in the Paris 2024 Olympic Games or sports eliminated from the Olympic programme. We split some sports into subdisciplines (e.g., athletics and distance running and marathon or walking; Alpine skiing and Nordic skiing; cycling and mountain biking) when their natures were too different and sufficient data were available.

**Table 1:** List of the journals.

**1. ACSM Health & Fitness Journal** **2. Adapted Physical Activity Quarterly** **3. American Journal of Physical Medicine & Rehabilitation** **4. American Journal of Sports Medicine** **5. Applied Physiology Nutrition and Metabolism** **6. Archives of Budo** **7. Archives of Physical Medicine and Rehabilitation** **8. Arthroscopy-The Journal of Arthroscopic and Related Surgery** **9. Biology of Sport** **10. BMC Sports Science Medicine and Rehabilitation** **11. British Journal of Sports Medicine** 12. British Medical Journal Open Sport Exercise 13. Canadian Journal of Applied Physiology **14. Clinical Biomechanics** **15. Clinical Journal of Sport Medicine** **16. Clinics in Sports Medicine** **17. Current Sports Medicine Reports** 18. Deutsche Zeitschrift fur Sportmedizin **19. European Journal of Applied Physiology** **20. European Journal of Sport Science** 21. European Sport Management Quarterly **22. Exercise and Sport Sciences Reviews** **23. Exercise Immunology Review** 24. Frontiers in Sports and Active Living **25. Gait & Posture** **26. High Altitude Medicine & Biology** **27. Human Movement Science** **28. International Journal of Performance Analysis In Sport** 29. International Journal of Sport Finance **30. International Journal of Sport Nutrition and Exercise Metabolism** **31. International Journal of Sport Psychology** 32. International Journal of Sports Marketing & Sponsorship **33. International Journal of Sports Medicine** **34. International Journal of Sports Physiology and Performance** 35. International Journal of Sports Science & Coaching **36**. International Journal of the History of Sport 37. International Review for The Sociology of Sport 38. International Review of Sport and Exercise Psychology **39. Isokinetics and Exercise Science** 40. Japanese Journal of Physical Fitness and Sports Medicine **41. Journal of Aging and Physical Activity** **42. Journal of Applied Biomechanics** **43. Journal of Applied Physiology** **44. Journal of Applied Sport Psychology** **45. Journal of Athletic Training** 46. Journal of Clinical Sport Psychology **47. Journal of Electromyography and Kinesiology** **48. Journal of Exercise Science & Fitness** 49. Journal of Hospitality Leisure Sport & Tourism Education **50. Journal of Human Kinetics** **51. Journal of Motor Behavior** **52. Journal of Orthopaedic & Sports Physical Therapy** **53. Journal of Orthopaedic Trauma** **54. Journal of Rehabilitation Medicine** **55. Journal of Science and Medicine in Sport** **56. Journal of Shoulder and Elbow Surgery** **57. Journal of Sport & Exercise Psychology** 58. Journal of Sport & Social Issues **59. Journal of Sport and Health Science** **60**. Journal of Sport History **61. Journal of Sport Management** **62. Journal of Sport Rehabilitation** 63. Journal of Sports Chiropractic & Rehabilitation 64. Journal of Sports Economics **65. Journal of Sports Medicine and Physical Fitness** **66. Journal of Sports Science and Medicine** **67. Journal of Sports Sciences** 68. Journal of Sports Traumatology and Related Research **69. Journal of Strength and Conditioning Research** **70. Journal of Teaching in Physical Education** **71. Journal of The International Society of Sports Nutrition** 72. Journal of The Philosophy of Sport **73. Kinesiology** **74. Knee** **75. Knee Surgery Sports Traumatology Arthroscopy** **76. Measurement in Physical Education and Exercise Science** **77. Medicina Dello Sport** **78. Medicine and Science in Sports and Exercise** **79. Motor Control** **80. Operative Techniques in Sports Medicine** **81. Orthopaedic Journal of Sports Medicine** **82. Pediatric Exercise Science** 83. Physical Education and Sport Pedagogy **84. Physical Therapy in Sport** **85. Physician and Sportsmedicine** **86. Physikalische Medizin Rehabilitationsmedizin Kurortmedizin** **87. PM&R** **88. Proceedings of The Institution of Mechanical Engineers Part P-Journal of Sports Engineering and Technology** **89. Psychology of Sport and Exercise** **90. Quest** **91. Research in Sports Medicine** **92. Research Quarterly for Exercise and Sport** 93. Research Quarterly for Exercise and Sport **94. Revista Brasileira De Medicina Do Esporte** **95. Revista Internacional De Medicina Y Ciencias De La Actividad Fisica Y Del Deporte** **96. Scandinavian Journal of Medicine & Science in Sports** **97. Science & Sports** **98. Sociology of Sport Journal** 99. South African Journal for Research in Sport Physical Education and Recreation **100. Sport Education and Society** 101. Sport Exercise and Performance Psychology 102. Sport in Society 103. Sport Management Review 104. Sport Marketing Quarterly **105. Sport Psychologist** 106. Sport Science Review 107. Sports (Basel) **108. Sports Biomechanics** 109. Sports Exercise and Injury **110. Sports Health-A Multidisciplinary Approach** **111. Sports Medicine** **112. Sports Medicine and Arthroscopy Review** **113. Sportverletzung-Sportschaden** **114. Strength and Conditioning Journal** **115. Wilderness & Environmental Medicine** 116. Zeitschrift fur Sportpsychologie

*The 85 journals of the Clarivate^TM^ Incites Journal Citation Reports “Sport Sciences” category are displayed in bold*.

*https://jcr.clarivate.com*.

**Table 2:** Summer **(A)** and Winter **(B)** Olympic sports (https://olympics.com/en/sports).

**A. SUMMER SPORTS**
**117. Archery 118. Athletics 119. Badminton 120. Baseball 121. Basketball 122. Boxing 123. Canoe-Kayak 124. Cycling 125. Diving 126. Equestrian 127. Fencing 128. Field Hockey 129. Football 130. Golf 131. Gymnastics 132. Handball 133. Judo 134. Karate 135. Marathon 136. Modern Pentathlon 137. Mountain Biking 138. Rowing 139. Rugby Sevens 140. Sailing 141. Shooting 142. Skateboarding 143. Softball 144. Sport Climbing 145. Surfing 146. Swimming 147. Table Tennis 148. Taekwondo 149. Tennis 150. Trampoline 151. Triathlon 152. Volleyball 153. Walking 154. Waterpolo 155. Weightlifting 156. Wrestling**
**B. WINTER SPORTS**
**157. Alpine—Freestyle Skiing 158. Biathlon 159. Bobsleigh 160. Curling 161. Ice Hockey 162. Luge 163. Nordic Skiing 164. Skating 165. Skeleton 166. Snowboard**

*“Marathon” and “Walking” (Athletics) as well as “Mountain biking” (Cycling) are displayed separately*.

*Skiing is displayed in 2 separated categories: “Alpine and freestyle skiing” and “Nordic skiing.”*

The search was performed on 4–5 June 2021 on article titles, and the inclusion and exclusion items are displayed in Table 3. Searching for only the sports or athletes (e.g., judo and judoka) in all these “sport sciences” journals would have yielded 103,164 articles, with many of them irrelevant in terms of our goals. By selecting only articles related to the selected sports—e.g., excluding animal, paralympic, and ultra-sports and fulfilling the inclusion and exclusion (e.g., “American football” for “football” or “water skiing” for “alpine skiing” or “athletes”) criteria (see Tables 3A,B for the specific criteria of each sport), we reduced the final number of articles to 25,003 (23,334 articles on summer sports and 1,669 on winter sports). If two different sports were mentioned in the article title, the article was allocated to both. All articles were double-checked (GPM and FB) for conformity with the selection criteria. Auto citations were not removed from this analysis.

**Table 3:** Inclusion and exclusion criteria in the search for **(A)** all sports, **(B)** the summer, and **(C)** winter Olympic sports.

**A. ALL SPORTS**				
**Exclusion topic**	**Exclusion items**			
Animal	Rats, mice, mouse, dog, cat, horse, fish			
Paralympic	Disabl#, paral#, wheelchair			
Ultra-sport	Ultra			
Retracted articles	Retract#			
**Sports**	**Inclusion items**	**Nb articles**	**Exclusion**	**Nb articles**
**B. SUMMER SPORTS**				
Archery	Archery, archer	43		43
Athletics	athletics, decathlon, decathlete, heptathlon, heptathlete, track and field, track-and-field javelin, shot put, shot-put, shot putter, high jump, long jump, discus throw, triple jump, pole vault, pole-vault, pole-vaulter, hammer throw, steeple chase, hurdle, hurdler, sprint, sprinter, sprinting, relay	8,492	Athlete, cycling, cyclist, swim, ski, skier, football, soccer, rugby, repeated-sprint	1,586
Badminton	Badminton	143		143
Baseball	Baseball	953		949
Basketball	Basketball, basket player	1,064		1,042
Boxing	Boxing, boxer	225		223
Canoe-Kayak	Canoe, kayak, canoeist, kayaker, kayakist, paddler	184		180
Cycling	Cycling, cyclist, bike, bicycle, bicycling, BMX	3,809	Triathlon, triathlete, mountain bike	3,550
Diving	Diving, diver, springboard	435	Breath-hold, scuba, apnea, football, pearl diver, decompression	52
Equestrian	Equestrian, horseman, horsemen, horse rider, horse-rider, horse riding, horse-riding, equitation	58		52
Fencing	Fencing, fencer	90		90
Field Hockey	Field hockey, hockey	166	ice	167
Football	Football, soccer, foot player, footballer	5,444	American football, league football, NFL, Gaelic football, Australian rules football, rugby football, quarterback	4,937
Golf	Golf, golfer	491		491
Gymnastics	Gymnastics, gymnastic, gymnast, floor exercise, horizontal bar, parallel bars, pommel horse, uneven bars, balance beam	429		428
Handball	Handball, handballer	440		440
Judo	Judo, judoka	262		261
Karate	Karate, karateka	114		113
Marathon—running	Marathon, marathoner, running, runner, middle-distance, long-distance	2,030	all sports but running	1,499
Modern pentathlon	Pentathlon, pentathlete	12		12
Mountain biking	Mountain bike, mountainbike, mountain biker	70		64
Rowing	Rowing, rower	678		673
Rugby sevens	Rugby sevens	89		89
Sailing	Sailing, sailer, sailor, windsurfing, windsurfer	110		109
Shooting	Shooting, shooter, riffle	135	football, soccer, handball, basketball	55
Skateboarding	Skateboarding, skateboarder	27		27
Softball	Softball	123		122
Sport Climbing	climbing, climber	512	step, stair, ladder, altitude, cyclist, cycling, mountaineer, mountaineering	338
Surfing	Surfing, surf, surfer	124	windsurf	100
Swimming	Swimming, swimmer, butterfly, backstroke, freestyle, free style, breaststroke, front crawl, frontcrawl, front-crawl	2,268		2,009
Table Tennis	Table tennis	90		90
Taekwondo	Taekwondo	159		159
Tennis	Tennis	1,054	Table tennis	954
Trampoline	Trampoline	43		41
Triathlon	Triathlon, triathlete	548	Ironman	425
Volleyball	Volleyball, volley-ball, volley ball, beach volley, volley player	606		602
Walking	Walking, walker	323		319
Waterpolo	Waterpolo, water polo, water-polo	150		136
Weightlifting	Weightlifting, weightlifter	264		264
Wrestling	Wrestling, wrestler	405		400
**C. WINTER SPORTS**				
Alpine—freestyle skiing	Alpine skiing, alpine ski, alpine skier, freestyle skiing, freestyle ski, freestyle skier, giant slalom, slalom	300	Canoe, kayak, water ski, water-ski	294
Biathlon	Biathlon, biathlete	47		47
Bobsleigh	Bobsleigh, bobsled	7		7
Curling	Curling, curler	8		8
Ice Hockey	Ice hockey, ice-hockey, NHL, National Hockey League	540		540
Luge	Luge	6		6
Nordic skiing	Cross-country ski, cross-country skier, cross-country skiing, Crosscountry ski, crosscountry skier, ski jumping, ski jumper, Nordic combined	369		369
Skating	Ice skating, ice skater, Ice-skating, ice-skater short track, skating, skate, figure skate, speed skating, speed skater	380	roller	334
Skeleton	Skeleton	17		12
Snowboard	Snowboard, snowboarding, snowboarder	152		152

*The number of articles found with the inclusion criteria and with the subsequent exclusion criteria and “manual cleaning” of the database are displayed*.

On 15 June, we performed a complete search for all these articles on Web of Science (Clarivate Analytics, USA). Basic information, including author(s), source journal, publication year, citations per year, and the total number of citations as well as keywords, was extracted. For each sport, the articles were listed based on citation frequency from highest to lowest, and the main metrics were averaged for the top 10 articles in each sport.

**Figure 1:**
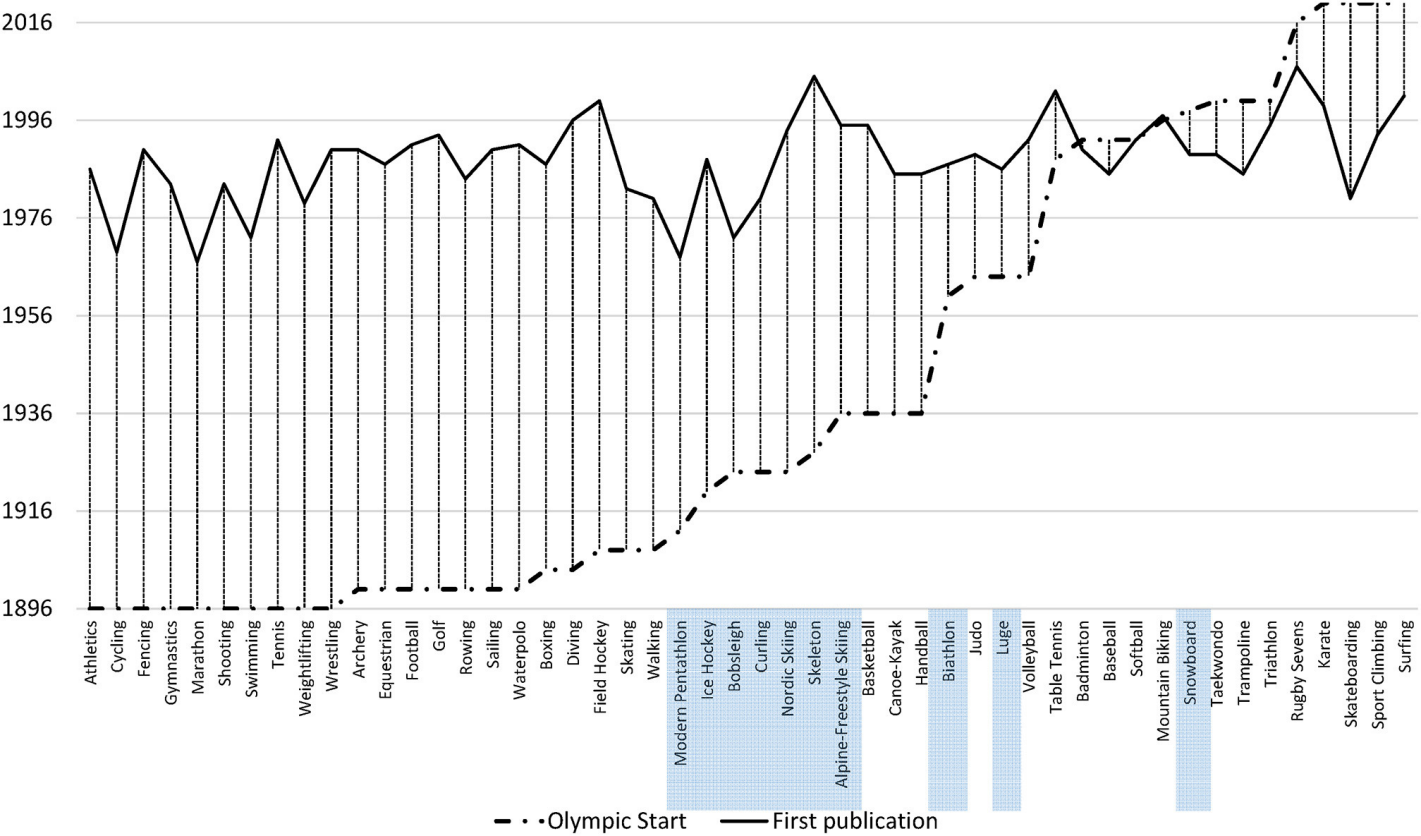
Dates of the Olympic debut and of first publication across all summer and winter Olympic sports. Winter sports are highlighted.

**Figure 2:**
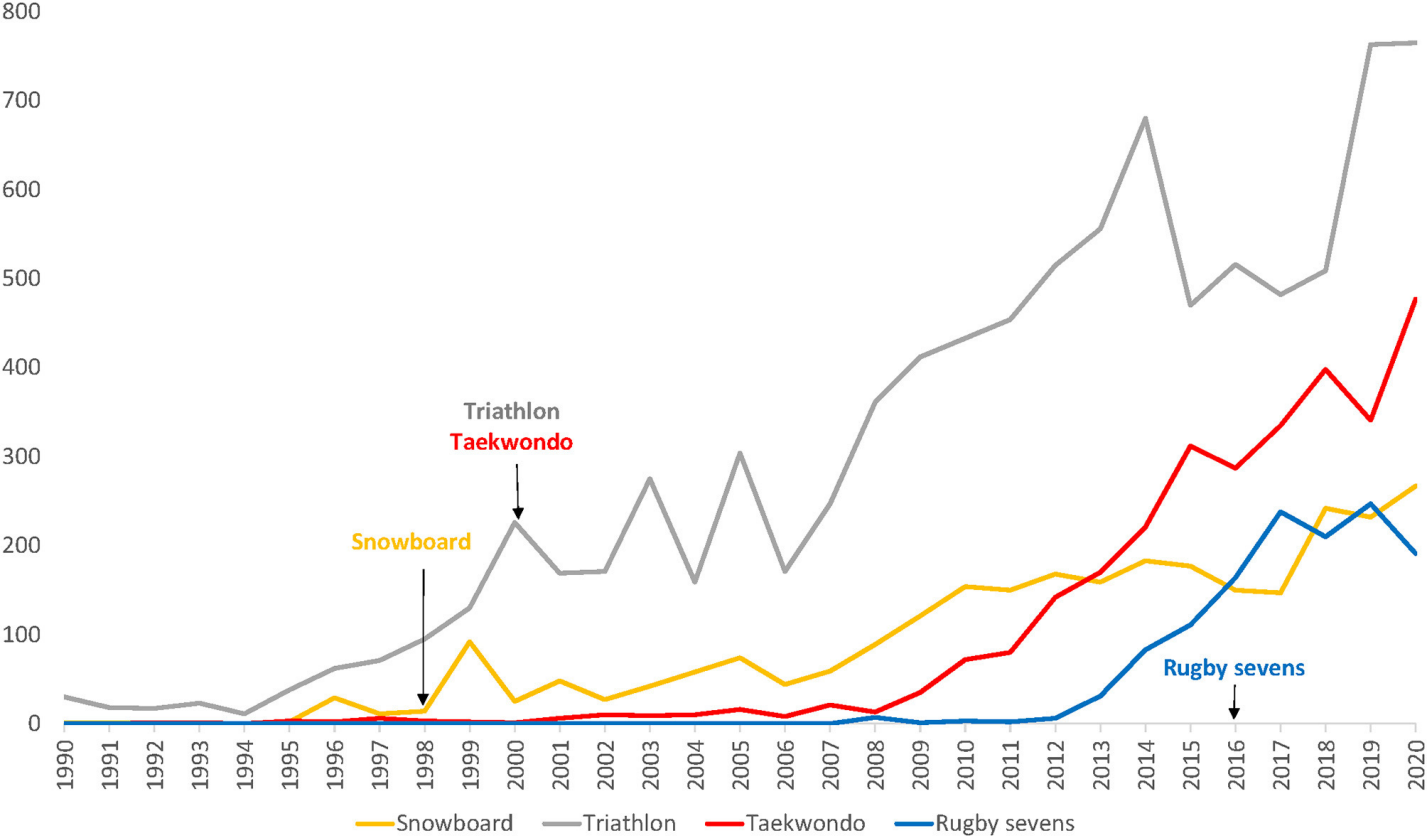
Number of citations per year (y-axis) in four “recent” (i.e., debut at 1998 or later) Olympic sports. The date of the Olympic debut is marked by an arrow.

We compared the dates of the Olympic debut and the first publication for each sport (Figure 1) and for the “recent” Olympic sports (i.e., with an Olympic debut in 1998 or later) to display the potential influence of being in the Olympic programme on the scientific interest in a sport (Figure 2).

We also compiled the keywords related to six main research topics [1. Physiology; 2. Performance; 3. Training and testing (i.e., fitness, testing, training); 4. Injuries and medicine (i.e., doping, injuries, medicine, rehabilitation); 5. Biomechanics (i.e., biomechanics, movement, motor control, equipment); 6. Psychology] for each sport. We display the top 5 most cited articles for every summer (Table 4) and winter (Table 5) Olympic sport.

**Table 4:** Top-5 articles on summer sports.

**References**	**Articles**	**Number citations**
**1. ARCHERY**		
Salazar et al. (1990)	Salazar W, Landers DM, Petruzzello SJ, Han M, Crews DJ, Kubitz KA. Hemispheric asymmetry, cardiac response, and performance in elite archers. Res Q Exerc Sport. 1990 Dec;61(4):351-9.	99
Landers et al. (1991)	Landers DM, Petruzzello SJ, Salazar W, Crews DJ, Kubitz KA, Gannon TL, et al. The influence of electrocortical biofeedback on performance in pre-elite archers. Med Sci Sports Exerc. 1991 Jan;23(1):123-9.	85
Ertan et al. (2003)	Ertan H, Kentel B, Tumer ST, Korkusuz F. Activation patterns in forearm muscles during archery shooting. Hum Mov Sci. 2003 Feb;22(1):37-45.	40
Leroyer et al. (1993)	Leroyer P, Van Hoecke J, Helal JN. Biomechanical study of the final push-pull in archery. J Sports Sci. 1993 Feb;11(1):63-9.	35
Mann and Littke (1989)	Mann DL, Littke N. Shoulder injuries in archery. Can J Sport Sci. 1989 Jun;14(2):85-92.	29
**2. ATHLETICS**		
Mero et al. (1992)	Mero A, Komi PV, Gregor RJ. Biomechanics of sprint running. A review. Sports Med. 1992 Jun;13(6):376-92.	363
Young et al. (1995)	Young W, McLean B, Ardagna J. Relationship between strength qualities and sprinting performance. J Sports Med Phys Fitness. 1995 Mar;35(1):13-9.	237
Hunter et al. (2005)	Hunter JP, Marshall RN, McNair PJ. Relationships between ground reaction force impulse and kinematics of sprint-running acceleration. J Appl Biomech. 2005 Feb;21(1):31-43.	217
Chelly and Denis (2001)	Chelly SM, Denis C. Leg power and hopping stiffness: relationship with sprint running performance. Med Sci Sports Exerc. 2001 Feb;33(2):326-33.	216
Kuitunen et al. (2002)	Kuitunen S, Komi PV, Kyrolainen H. Knee and ankle joint stiffness in sprint running. Med Sci Sports Exerc. 2002 Jan;34(1):166-73.	214
**3. BADMINTON**		
Cabello Manrique and Gonzalez-Badillo (2003)	Cabello Manrique D, Gonzalez-Badillo JJ. Analysis of the characteristics of competitive badminton. Br J Sports Med. 2003 Feb;37(1):62-6.	121
Phomsoupha and Laffaye (2015)	Phomsoupha M, Laffaye G. The science of badminton: game characteristics, anthropometry, physiology, visual fitness and biomechanics. Sports Med. 2015 Apr;45(4):473-95.	83
Callow et al. (2001)	Callow N, Hardy L, Hall C. The effects of a motivational general-mastery imagery intervention on the sport confidence of high-level badminton players. Res Q Exerc Sport. 2001 Dec;72(4):389-400.	81
Faude et al. (2007)	Faude O, Meyer T, Rosenberger F, Fries M, Huber G, Kindermann W. Physiological characteristics of badminton match play. Eur J Appl Physiol. 2007 Jul;100(4):479-85.	71
Kuntze et al. (2010)	Kuntze G, Mansfield N, Sellers W. A biomechanical analysis of common lunge tasks in badminton. J Sports Sci. 2010 Jan;28(2):183-91.	59
**4. BASEBALL**		
Fleisig et al. (1995)	Fleisig GS, Andrews JR, Dillman CJ, Escamilla RF. Kinetics of baseball pitching with implications about injury mechanisms. Am J Sports Med. 1995 Mar-Apr;23(2):233-9.	776
Lyman et al. (2002)	Lyman S, Fleisig GS, Andrews JR, Osinski ED. Effect of pitch type, pitch count, and pitching mechanics on risk of elbow and shoulder pain in youth baseball pitchers. Am J Sports Med. 2002 Jul-Aug;30(4):463-8.	391
Crockett et al. (2002)	Crockett HC, Gross LB, Wilk KE, Schwartz ML, Reed J, O'Mara J, et al. Osseous adaptation and range of motion at the glenohumeral joint in professional baseball pitchers. Am J Sports Med. 2002 Jan-Feb;30(1):20-6.	373
Olsen et al. (2006)	Olsen SJ, 2nd, Fleisig GS, Dun S, Loftice J, Andrews JR. Risk factors for shoulder and elbow injuries in adolescent baseball pitchers. Am J Sports Med. 2006 Jun;34(6):905-12.	359
Fleisig et al. (1999)	Fleisig GS, Barrentine SW, Zheng N, Escamilla RF, Andrews JR. Kinematic and kinetic comparison of baseball pitching among various levels of development. J Biomech. 1999 Dec;32(12):1371-5.	336
**5. BASKETBALL**		
Arendt and Dick (1995)	Arendt E, Dick R. Knee injury patterns among men and women in collegiate basketball and soccer. NCAA data and review of literature. Am J Sports Med. 1995 Nov-Dec;23(6):694-701.	1012
Plisky et al. (2006)	Plisky PJ, Rauh MJ, Kaminski TW, Underwood FB. Star Excursion Balance Test as a predictor of lower extremity injury in high school basketball players. J Orthop Sports Phys Ther. 2006 Dec;36(12):911-9.	591
Krosshaug et al. (2007)	Krosshaug T, Nakamae A, Boden BP, Engebretsen L, Smith G, Slauterbeck JR, et al. Mechanisms of anterior cruciate ligament injury in basketball: video analysis of 39 cases. Am J Sports Med. 2007 Mar;35(3):359-67.	587
Ford et al. (2003)	Ford KR, Myer GD, Hewett TE. Valgus knee motion during landing in high school female and male basketball players. Med Sci Sports Exerc. 2003 Oct;35(10):1745-50.	557
Agel et al. (2005)	Agel J, Arendt EA, Bershadsky B. Anterior cruciate ligament injury in national collegiate athletic association basketball and soccer: a 13-year review. Am J Sports Med. 2005 Apr;33(4):524-30.	491
**6. BOXING**		
Walilko et al. (2005)	Walilko TJ, Viano DC, Bir CA. Biomechanics of the head for Olympic boxer punches to the face. Br J Sports Med. 2005 Oct;39(10):710-9.	158
Hall and Lane (2001)	Hall CJ, Lane AM. Effects of rapid weight loss on mood and performance among amateur boxers. Br J Sports Med. 2001 Dec;35(6):390-5.	99
Otto et al. (2000)	Otto M, Holthusen S, Bahn E, Sohnchen N, Wiltfang J, Geese R, et al. Boxing and running lead to a rise in serum levels of S-100B protein. Int J Sports Med. 2000 Nov;21(8):551-5.	95
Smith et al. (2000)	Smith MS, Dyson RJ, Hale T, Janaway L. Development of a boxing dynamometer and its punch force discrimination efficacy. J Sports Sci. 2000 Jun;18(6):445-50.	95
Hristovski et al. (2006)	Hristovski R, Davids K, Araujo D, Button C. How boxers decide to punch a target: emergent behaviour in nonlinear dynamical movement systems. J Sports Sci Med. 2006; 5(CSSI):60-73.	82
**7. CANOE-KAYAK**		
Bishop et al. (2002)	Bishop D, Bonetti D, Dawson B. The influence of pacing strategy on VO2 and supramaximal kayak performance. Med Sci Sports Exerc. 2002 Jun;34(6):1041-7.	145
Mackinnon et al. (1993)	Mackinnon LT, Ginn E, Seymour GJ. Decreased salivary immunoglobulin A secretion rate after intense interval exercise in elite kayakers. Eur J Appl Physiol Occup Physiol. 1993;67(2):180-4.	113
Liow and Hopkins (2003)	Liow DK, Hopkins WG. Velocity specificity of weight training for kayak sprint performance. Med Sci Sports Exerc. 2003 Jul;35(7):1232-7.	90
Garcia-Pallares et al. (2009)	Garcia-Pallares J, Sanchez-Medina L, Carrasco L, Diaz A, Izquierdo M. Endurance and neuromuscular changes in world-class level kayakers during a periodized training cycle. Eur J Appl Physiol. 2009 Jul;106(4):629-38.	79
Ackland et al. (2003)	Ackland TR, Ong KB, Kerr DA, Ridge B. Morphological characteristics of Olympic sprint canoe and kayak paddlers. J Sci Med Sport. 2003 Sep;6(3):285-94.	75
**8. CYCLING**		
Coyle et al. (1992)	Coyle EF, Sidossis LS, Horowitz JF, Beltz JD. Cycling efficiency is related to the percentage of type I muscle fibers. Med Sci Sports Exerc. 1992 Jul;24(7):782-8.	418
Oja et al. (2011)	Oja P, Titze S, Bauman A, de Geus B, Krenn P, Reger-Nash B, et al. Health benefits of cycling: a systematic review. Scand J Med Sci Sports. 2011 Aug;21(4):496-509.	412
Coyle et al. (1991)	Coyle EF, Feltner ME, Kautz SA, Hamilton MT, Montain SJ, Baylor AM, et al. Physiological and biomechanical factors associated with elite endurance cycling performance. Med Sci Sports Exerc. 1991 Jan;23(1):93-107.	380
Bassett et al. (2008)	Bassett DR, Jr., Pucher J, Buehler R, Thompson DL, Crouter SE. Walking, cycling, and obesity rates in Europe, North America, and Australia. J Phys Act Health. 2008 Nov;5(6):795-814.	362
Hermansen and Saltin (1969)	Hermansen L, Saltin B. Oxygen uptake during maximal treadmill and bicycle exercise. J Appl Physiol. 1969 Jan;26(1):31-7.	354
**9. DIVING**		
Baranto et al. (2006)	Baranto A, Hellstrom M, Nyman R, Lundin O, Sward L. Back pain and degenerative abnormalities in the spine of young elite divers: a 5-year follow-up magnetic resonance imaging study. Knee Surg Sports Traumatol Arthrosc. 2006 Sep;14(9):907-14.	43
Blanksby et al. (1997)	Blanksby BA, Wearne FK, Elliott BC, Blitvich JD. Aetiology and occurrence of diving injuries. A review of diving safety. Sports Med. 1997 Apr;23(4):228-46.	39
Schmitt and Gerner (2001)	Schmitt H, Gerner HJ. Paralysis from sport and diving accidents. Clin J Sport Med. 2001 Jan;11(1):17-22.	37
Lewis et al. (2013)	Lewis RM, Redzic M, Thomas DT. The effects of season-long vitamin D supplementation on collegiate swimmers and divers. Int J Sport Nutr Exerc Metab. 2013 Oct;23(5):431-40.	35
Barris et al. (2014)	Barris S, Farrow D, Davids K. Increasing functional variability in the preparatory phase of the takeoff improves elite springboard diving performance. Res Q Exerc Sport. 2014 Mar;85(1):97-106.	32
**10. EQUESTRIAN**		
Paix (1999)	Paix BR. Rider injury rates and emergency medical services at equestrian events. Br J Sports Med. 1999 Feb;33(1):46-8.	59
Devienne and Guezennec (2000)	Devienne MF, Guezennec CY. Energy expenditure of horse riding. Eur J Appl Physiol. 2000 Aug;82(5-6):499-503.	51
Lloyd (1987)	Lloyd RG. Riding and other equestrian injuries: considerable severity. Br J Sports Med. 1987 Mar;21(1):22-4.	51
McCrory and Turner (2005)	McCrory P, Turner M. Equestrian injuries. Med Sport Sci. 2005;48:8-17.	45
Kusma et al. (2004)	Kusma M, Jung J, Dienst M, Goedde S, Kohn D, Seil R. Arthroscopic treatment of an avulsion fracture of the ligamentum teres of the hip in an 18-year-old horse rider. Arthroscopy. 2004 Jul;20 Suppl 2:64-6.	37
**11. FENCING**		
Roi and Bianchedi (2008)	Roi GS, Bianchedi D. The science of fencing: implications for performance and injury prevention. Sports Med. 2008;38(6):465-81.	107
Giombini et al. (2013)	Giombini A, Dragoni S, Di Cesare A, Di Cesare M, Del Buono A, Maffulli N. Asymptomatic Achilles, patellar, and quadriceps tendinopathy: a longitudinal clinical and ultrasonographic study in elite fencers. Scand J Med Sci Sports. 2013 Jun;23(3):311-6.	48
Hosseini and Lifshitz (2009)	Hosseini AH, Lifshitz J. Brain injury forces of moderate magnitude elicit the fencing response. Med Sci Sports Exerc. 2009 Sep;41(9):1687-97.	48
Taddei et al. (2012)	Taddei F, Bultrini A, Spinelli D, Di Russo F. Neural correlates of attentional and executive processing in middle-age fencers. Med Sci Sports Exerc. 2012 Jun;44(6):1057-66.	46
Williams and Walmsley (2000)	Williams LR, Walmsley A. Response timing and muscular coordination in fencing: a comparison of elite and novice fencers. J Sci Med Sport. 2000 Dec;3(4):460-75.	41
**12. FIELD HOCKEY**		
Spencer et al. (2004)	Spencer M, Lawrence S, Rechichi C, Bishop D, Dawson B, Goodman C. Time-motion analysis of elite field hockey, with special reference to repeated-sprint activity. J Sports Sci. 2004 Sep;22(9):843-50.	275
Cochrane and Stannard (2005)	Cochrane DJ, Stannard SR. Acute whole body vibration training increases vertical jump and flexibility performance in elite female field hockey players. Br J Sports Med. 2005 Nov;39(11):860-5.	254
MacLeod et al. (2009)	MacLeod H, Morris J, Nevill A, Sunderland C. The validity of a non-differential global positioning system for assessing player movement patterns in field hockey. J Sports Sci. 2009 Jan 15;27(2):121-8.	133
Aziz et al. (2000)	Aziz AR, Chia M, Teh KC. The relationship between maximal oxygen uptake and repeated sprint performance indices in field hockey and soccer players. J Sports Med Phys Fitness. 2000 Sep;40(3):195-200.	111
Elferink-Gemser et al. (2004)	Elferink-Gemser MT, Visscher C, Lemmink KA, Mulder TW. Relation between multidimensional performance characteristics and level of performance in talented youth field hockey players. J Sports Sci. 2004 Nov-Dec;22(11-12):1053-63.	103
**13. FOOTBALL**		
Stolen et al. (2005)	Stolen T, Chamari K, Castagna C, Wisloff U. Physiology of soccer: an update. Sports Med. 2005;35(6):501-36.	1150
Mohr et al. (2003)	Mohr M, Krustrup P, Bangsbo J. Match performance of high-standard soccer players with special reference to development of fatigue. J Sports Sci. 2003 Jul;21(7):519-28.	1120
Arendt and Dick (1995)	Arendt E, Dick R. Knee injury patterns among men and women in collegiate basketball and soccer. NCAA data and review of literature. Am J Sports Med. 1995 Nov-Dec;23(6):694-701.	1013
Reilly et al. (2000)	Reilly T, Bangsbo J, Franks A. Anthropometric and physiological predispositions for elite soccer. J Sports Sci. 2000 Sep;18(9):669-83.	781
Impellizzeri et al. (2004)	Impellizzeri FM, Rampinini E, Coutts AJ, Sassi A, Marcora SM. Use of RPE-based training load in soccer. Med Sci Sports Exerc. 2004 Jun;36(6):1042-7.	635
**14. GOLF**		
Wulf et al. (1999)	Wulf G, Lauterbach B, Toole T. The learning advantages of an external focus of attention in golf. Res Q Exerc Sport. 1999 Jun;70(2):120-6.	258
Wulf and Su (2007)	Wulf G, Su J. An external focus of attention enhances golf shot accuracy in beginners and experts. Res Q Exerc Sport. 2007 Sep;78(4):384-9.	233
Hume et al. (2005)	Hume PA, Keogh J, Reid D. The role of biomechanics in maximising distance and accuracy of golf shots. Sports Med. 2005;35(5):429-49.	163
Perkins-Ceccato et al. (2003)	Perkins-Ceccato N, Passmore SR, Lee TD. Effects of focus of attention depend on golfers' skill. J Sports Sci. 2003 Aug;21(8):593-600.	138
Vad et al. (2004)	Vad VB, Bhat AL, Basrai D, Gebeh A, Aspergren DD, Andrews JR. Low back pain in professional golfers: the role of associated hip and low back range-of-motion deficits. Am J Sports Med. 2004 Mar;32(2):494-7.	124
**15. GYMNASTICS**		
Bressel et al. (2007)	Bressel E, Yonker JC, Kras J, Heath EM. Comparison of static and dynamic balance in female collegiate soccer, basketball, and gymnastics athletes. J Athl Train. 2007 Jan-Mar;42(1):42-6.	221
Kolt and Kirkby (1999)	Kolt GS, Kirkby RJ. Epidemiology of injury in elite and subelite female gymnasts: a comparison of retrospective and prospective findings. Br J Sports Med. 1999 Oct;33(5):312-8.	140
Bencke et al. (2002)	Bencke J, Damsgaard R, Saekmose A, Jorgensen P, Jorgensen K, Klausen K. Anaerobic power and muscle strength characteristics of 11 years old elite and non-elite boys and girls from gymnastics, team handball, tennis and swimming. Scand J Med Sci Sports. 2002 Jun;12(3):171-8.	137
Cassell et al. (1996)	Cassell C, Benedict M, Specker B. Bone mineral density in elite 7- to 9-yr-old female gymnasts and swimmers. Med Sci Sports Exerc. 1996 Oct;28(10):1243-6.	134
Caine et al. (1989)	Caine D, Cochrane B, Caine C, Zemper E. An epidemiologic investigation of injuries affecting young competitive female gymnasts. Am J Sports Med. 1989 Nov-Dec;17(6):811-20.	132
**16. HANDBALL**		
Olsen et al. (2004)	Olsen OE, Myklebust G, Engebretsen L, Bahr R. Injury mechanisms for anterior cruciate ligament injuries in team handball: a systematic video analysis. Am J Sports Med. 2004 Jun;32(4):1002-12.	711
Myklebust et al. (2003)	Myklebust G, Engebretsen L, Braekken IH, Skjolberg A, Olsen OE, Bahr R. Prevention of anterior cruciate ligament injuries in female team handball players: a prospective intervention study over three seasons. Clin J Sport Med. 2003 Mar;13(2):71-8.	504
Koga et al. (2010)	Koga H, Nakamae A, Shima Y, Iwasa J, Myklebust G, Engebretsen L, et al. Mechanisms for noncontact anterior cruciate ligament injuries: knee joint kinematics in 10 injury situations from female team handball and basketball. Am J Sports Med. 2010 Nov;38(11):2218-25.	368
Myklebust et al. (1998)	Myklebust G, Maehlum S, Holm I, Bahr R. A prospective cohort study of anterior cruciate ligament injuries in elite Norwegian team handball. Scand J Med Sci Sports. 1998 Jun;8(3):149-53.	283
Gorostiaga et al. (2005)	Gorostiaga EM, Granados C, Ibanez J, Izquierdo M. Differences in physical fitness and throwing velocity among elite and amateur male handball players. Int J Sports Med. 2005 Apr;26(3):225-32.	231
**17. JUDO**		
Franchini et al. (2011)	Franchini E, Del Vecchio FB, Matsushigue KA, Artioli GG. Physiological profiles of elite judo athletes. Sports Med. 2011 Feb 1;41(2):147-66.	291
Perrin et al. (2002)	Perrin P, Deviterne D, Hugel F, Perrot C. Judo, better than dance, develops sensorimotor adaptabilities involved in balance control. Gait Posture. 2002 Apr;15(2):187-94.	235
Degoutte et al. (2003)	Degoutte F, Jouanel P, Filaire E. Energy demands during a judo match and recovery. Br J Sports Med. 2003 Jun;37(3):245-9.	133
Artioli et al. (2010)	Artioli GG, Franchini E, Nicastro H, Sterkowicz S, Solis MY, Lancha AH, Jr. The need of a weight management control program in judo: a proposal based on the successful case of wrestling. J Int Soc Sports Nutr. 2010 May 4;7:15.	129
Degoutte et al. (2006)	Degoutte F, Jouanel P, Begue RJ, Colombier M, Lac G, Pequignot JM, et al. Food restriction, performance, biochemical, psychological, and endocrine changes in judo athletes. Int J Sports Med. 2006 Jan;27(1):9-18.	121
**18. KARATE**		
Mori et al. (2002)	Mori S, Ohtani Y, Imanaka K. Reaction times and anticipatory skills of karate athletes. Hum Mov Sci. 2002 Jul;21(2):213-30.	170
Beneke et al. (2004)	Beneke R, Beyer T, Jachner C, Erasmus J, Hutler M. Energetics of karate kumite. Eur J Appl Physiol. 2004 Aug;92(4-5):518-23.	127
Chaabene et al. (2012)	Chaabene H, Hachana Y, Franchini E, Mkaouer B, Chamari K. Physical and physiological profile of elite karate athletes. Sports Med. 2012 Oct 1;42(10):829-43.	118
Doria et al. (2009)	Doria C, Veicsteinas A, Limonta E, Maggioni MA, Aschieri P, Eusebi F, et al. Energetics of karate (kata and kumite techniques) in top-level athletes. Eur J Appl Physiol. 2009 Nov;107(5):603-10.	81
Wong del et al. (2010)	Wong del P, Tan EC, Chaouachi A, Carling C, Castagna C, Bloomfield J, et al. Using squat testing to predict training loads for lower-body exercises in elite karate athletes. J Strength Cond Res. 2010 Nov;24(11):3075-80.	76
**19. MARATHON - LONG DISTANCE RUNNING**		
van Gent et al. (2007)	van Gent RN, Siem D, van Middelkoop M, van Os AG, Bierma-Zeinstra SM, Koes BW. Incidence and determinants of lower extremity running injuries in long distance runners: a systematic review. Br J Sports Med. 2007 Aug;41(8):469-80; discussion 80.	700
Conley and Krahenbuhl (1980)	Conley DL, Krahenbuhl GS. Running economy and distance running performance of highly trained athletes. Med Sci Sports Exerc. 1980;12(5):357-60.	455
Sjodin and Jacobs (1981)	Sjodin B, Jacobs I. Onset of blood lactate accumulation and marathon running performance. Int J Sports Med. 1981 Feb;2(1):23-6.	395
Nieman et al. (1990)	Nieman DC, Johanssen LM, Lee JW, Arabatzis K. Infectious episodes in runners before and after the Los Angeles Marathon. J Sports Med Phys Fitness. 1990 Sep;30(3):316-28.	373
Billat (2001)	Billat LV. Interval training for performance: a scientific and empirical practice. Special recommendations for middle- and long-distance running. Part I: aerobic interval training. Sports Med. 2001;31(1):13-31.	285
**20. MODERN PENTATHLON**		
Coutinho et al. (2016)	Coutinho LA, Porto CP, Pierucci AP. Critical evaluation of food intake and energy balance in young modern pentathlon athletes: a cross-sectional study. J Int Soc Sports Nutr. 2016;13:15.	14
Le Meur et al. (2010)	Le Meur Y, Hausswirth C, Abbiss C, Baup Y, Dorel S. Performance factors in the new combined event of modern pentathlon. J Sports Sci. 2010 Aug;28(10):1111-6.	12
Krahenbuhl et al. (1979)	Krahenbuhl GS, Wells CL, Brown CH, Ward PE. Characteristics of national and world class female pentathletes. Med Sci Sports. 1979 Spring;11(1):20-3.	11
Le Meur et al. (2012)	Le Meur Y, Dorel S, Baup Y, Guyomarch JP, Roudaut C, Hausswirth C. Physiological demand and pacing strategy during the new combined event in elite pentathletes. Eur J Appl Physiol. 2012 Jul;112(7):2583-93.	9
Dadswell et al. (2013)	Dadswell CE, Payton C, Holmes P, Burden A. Biomechanical analysis of the change in pistol shooting format in modern pentathlon. J Sports Sci. 2013;31(12):1294-301.	7
**21. MOUNTAIN BIKING**		
Lee et al. (2002)	Lee H, Martin DT, Anson JM, Grundy D, Hahn AG. Physiological characteristics of successful mountain bikers and professional road cyclists. J Sports Sci. 2002 Dec;20(12):1001-8.	70
Stapelfeldt et al. (2004)	Stapelfeldt B, Schwirtz A, Schumacher YO, Hillebrecht M. Workload demands in mountain bike racing. Int J Sports Med. 2004 May;25(4):294-300.	64
Gregory et al. (2007)	Gregory J, Johns DP, Walls JT. Relative vs. absolute physiological measures as predictors of mountain bike cross-country race performance. J Strength Cond Res. 2007 Feb;21(1):17-22.	49
MacRae et al. (2000)	MacRae H-H, Hise KJ, Allen PJ. Effects of front and dual suspension mountain bike systems on uphill cycling performance. Med Sci Sports Exerc. 2000 Jul;32(7):1276-80.	30
Seifert et al. (1997)	Seifert JG, Luetkemeier MJ, Spencer MK, Miller D, Burke ER. The effects of mountain bike suspension systems on energy expenditure, physical exertion, and time trial performance during mountain bicycling. Int J Sports Med. 1997 Apr;18(3):197-200.	26
**22. ROWING**		
Volianitis et al. (2001)	Volianitis S, McConnell AK, Koutedakis Y, McNaughton L, Backx K, Jones DA. Inspiratory muscle training improves rowing performance. Med Sci Sports Exerc. 2001 May;33(5):803-9.	206
Beneke (1995)	Beneke R. Anaerobic threshold, individual anaerobic threshold, and maximal lactate steady state in rowing. Med Sci Sports Exerc. 1995 Jun;27(6):863-7.	160
Bruce et al. (2000)	Bruce CR, Anderson ME, Fraser SF, Stepto NK, Klein R, Hopkins WG, et al. Enhancement of 2000-m rowing performance after caffeine ingestion. Med Sci Sports Exerc. 2000 Nov;32(11):1958-63.	151
Hagerman (1984)	Hagerman FC. Applied physiology of rowing. Sports Med. 1984 Jul-Aug;1(4):303-26.	149
Secher (1993)	Secher NH. Physiological and biomechanical aspects of rowing. Implications for training. Sports Med. 1993 Jan;15(1):24-42.	147
**23. RUGBY SEVENS**		
Higham et al. (2012)	Higham DG, Pyne DB, Anson JM, Eddy A. Movement patterns in rugby sevens: effects of tournament level, fatigue and substitute players. J Sci Med Sport. 2012 May;15(3):277-82.	106
Suarez-Arrones et al. (2014)	Suarez-Arrones L, Arenas C, Lopez G, Requena B, Terrill O, Mendez-Villanueva A. Positional differences in match running performance and physical collisions in men rugby sevens. Int J Sports Physiol Perform. 2014 Mar;9(2):316-23.	62
Higham et al. (2013)	Higham DG, Pyne DB, Anson JM, Eddy A. Physiological, anthropometric, and performance characteristics of rugby sevens players. Int J Sports Physiol Perform. 2013 Jan;8(1):19-27.	61
Suarez-Arrones et al. (2013)	Suarez-Arrones L, Calvo-Lluch A, Portillo J, Sanchez F, Mendez-Villanueva A. Running demands and heart rate response in rugby sevens referees. J Strength Cond Res. 2013 Jun;27(6):1618-22.	52
Takahashi et al. (2007)	Takahashi I, Umeda T, Mashiko T, Chinda D, Oyama T, Sugawara K, et al. Effects of rugby sevens matches on human neutrophil-related non-specific immunity. Br J Sports Med. 2007 Jan;41(1):13-8.	49
**24. SAILING**		
Saury and Durand (1998)	Saury J, Durand M. Practical knowledge in expert coaches: on-site study of coaching in sailing. Res Q Exerc Sport. 1998 Sep;69(3):254-66.	131
Allen (1999)	Allen JB. Sports medicine and sailing. Phys Med Rehabil Clin N Am. 1999 Feb;10(1):49-65.	52
Vogiatzis et al. (1995)	Vogiatzis I, Spurway NC, Wilson J, Boreham C. Assessment of aerobic and anaerobic demands of dinghy sailing at different wind velocities. J Sports Med Phys Fitness. 1995 Jun;35(2):103-7.	46
Aagaard et al. (1998)	Aagaard P, Beyer N, Simonsen EB, Larsson B, Magnusson SP, Kjaer M. Isokinetic muscle strength and hiking performance in elite sailors. Scand J Med Sci Sports. 1998 Jun;8(3):138-44.	39
Nathanson and Reinert (1999)	Nathanson AT, Reinert SE. Windsurfing injuries: results of a paper- and Internet-based survey. Wilderness Environ Med. 1999 Winter;10(4):218-25.	39
**25. SHOOTING**		
Era et al. (1996)	Era P, Konttinen N, Mehto P, Saarela P, Lyytinen H. Postural stability and skilled performance–a study on top-level and naive rifle shooters. J Biomech. 1996 Mar;29(3):301-6.	124
Mononen et al. (2007)	Mononen K, Konttinen N, Viitasalo J, Era P. Relationships between postural balance, rifle stability and shooting accuracy among novice rifle shooters. Scand J Med Sci Sports. 2007 Apr;17(2):180-5.	90
Causer et al. (2010)	Causer J, Bennett SJ, Holmes PS, Janelle CM, Williams AM. Quiet eye duration and gun motion in elite shotgun shooting. Med Sci Sports Exerc. 2010 Aug;42(8):1599-608.	74
Loze et al. (2001)	Loze GM, Collins D, Holmes PS. Pre-shot EEG alpha-power reactivity during expert air-pistol shooting: a comparison of best and worst shots. J Sports Sci. 2001 Sep;19(9):727-33.	65
Di Russo et al. (2005)	Di Russo F, Pitzalis S, Aprile T, Spinelli D. Effect of practice on brain activity: an investigation in top-level rifle shooters. Med Sci Sports Exerc. 2005 Sep;37(9):1586-93.	63
**26. SKATEBOARDING**		
Banas et al. (1992)	Banas MP, Dalldorf PG, Marquardt JD. Skateboard and in-line skate fractures: a report of one summer's experience. J Orthop Trauma. 1992;6(3):301-5.	
Fountain and Meyers (1996)	Fountain JL, Meyers MC. Skateboarding injuries. Sports Med. 1996 Dec;22(6):360-6.	
Forsman and Eriksson (2001)	Forsman L, Eriksson A. Skateboarding injuries of today. Br J Sports Med. 2001 Oct;35(5):325-8.	
Zalavras et al. (2005)	Zalavras C, Nikolopoulou G, Essin D, Manjra N, Zionts LE. Pediatric fractures during skateboarding, roller skating, and scooter riding. Am J Sports Med. 2005 Apr;33(4):568-73.	
Kroncke et al. (2008)	Kroncke EL, Niedfeldt MW, Young CC. Use of protective equipment by adolescents in inline skating, skateboarding, and snowboarding. Clin J Sport Med. 2008 Jan;18(1):38-43.	
**27. SOFTBALL**		
Shanley et al. (2012)	Shanley E, Michener LA, Ellenbecker TS, Rauh MJ. Shoulder range of motion, pitch count, and injuries among interscholastic female softball pitchers: a descriptive study. Int J Sports Phys Ther. 2012 Oct;7(5):548-57.	166
Nimphius et al. (2010)	Nimphius S, McGuigan MR, Newton RU. Relationship between strength, power, speed, and change of direction performance of female softball players. J Strength Cond Res. 2010 Apr;24(4):885-95.	111
Barrentine et al. (1998)	Barrentine SW, Fleisig GS, Whiteside JA, Escamilla RF, Andrews JR. Biomechanics of windmill softball pitching with implications about injury mechanisms at the shoulder and elbow. J Orthop Sports Phys Ther. 1998 Dec;28(6):405-15.	75
Marshall et al. (2007)	Marshall SW, Hamstra-Wright KL, Dick R, Grove KA, Agel J. Descriptive epidemiology of collegiate women's softball injuries: National Collegiate Athletic Association Injury Surveillance System, 1988-1989 through 2003-2004. J Athl Train. 2007 Apr-Jun;42(2):286-94.	66
Werner et al. (2006)	Werner SL, Jones DG, Guido JA, Jr., Brunet ME. Kinematics and kinetics of elite windmill softball pitching. Am J Sports Med. 2006 Apr;34(4):597-603.	62
**28. SPORT CLIMBING**		
Watts (2004)	Watts PB. Physiology of difficult rock climbing. Eur J Appl Physiol. 2004 Apr;91(4):361-72.	135
Mermier et al. (2000)	Mermier CM, Janot JM, Parker DL, Swan JG. Physiological and anthropometric determinants of sport climbing performance. Br J Sports Med. 2000 Oct;34(5):359-65; discussion 66.	113
Grant et al. (1996)	Grant S, Hynes V, Whittaker A, Aitchison T. Anthropometric, strength, endurance and flexibility characteristics of elite and recreational climbers. J Sports Sci. 1996 Aug;14(4):301-9.	107
Watts et al. (1993)	Watts PB, Martin DT, Durtschi S. Anthropometric profiles of elite male and female competitive sport rock climbers. J Sports Sci. 1993 Apr;11(2):113-7.	103
Billat et al. (1995)	Billat V, Palleja P, Charlaix T, Rizzardo P, Janel N. Energy specificity of rock climbing and aerobic capacity in competitive sport rock climbers. J Sports Med Phys Fitness. 1995 Mar;35(1):20-4.	101
**29. SURFING**		
Mendez-Villanueva et al. (2006)	Mendez-Villanueva A, Bishop D, Hamer P. Activity profile of world-class professional surfers during competition: a case study. J Strength Cond Res. 2006 Aug;20(3):477-82.	66
Nathanson et al. (2007)	Nathanson A, Bird S, Dao L, Tam-Sing K. Competitive surfing injuries: a prospective study of surfing-related injuries among contest surfers. Am J Sports Med. 2007 Jan;35(1):113-7.	57
Farley et al. (2012)	Farley OR, Harris NK, Kilding AE. Physiological demands of competitive surfing. J Strength Cond Res. 2012 Jul;26(7):1887-96.	57
Booth (2001)	Booth D. From bikinis to boardshorts: wahines and the paradoxes of surfing culture. J Sport Hist. 2001;28(1):3-22.	56
Furness et al. (2015)	Furness J, Hing W, Walsh J, Abbott A, Sheppard JM, Climstein M. Acute injuries in recreational and competitive surfers: incidence, severity, location, type, and mechanism. Am J Sports Med. 2015 May;43(5):1246-54.	39
**30. SWIMMING**		
Costill et al. (1985)	Costill DL, Kovaleski J, Porter D, Kirwan J, Fielding R, King D. Energy expenditure during front crawl swimming: predicting success in middle-distance events. Int J Sports Med. 1985 Oct;6(5):266-70.	287
Craig and Pendergast (1979)	Craig AB, Jr., Pendergast DR. Relationships of stroke rate, distance per stroke, and velocity in competitive swimming. Med Sci Sports. 1979 Fall;11(3):278-83.	230
Costill et al. (1988)	Costill DL, Flynn MG, Kirwan JP, Houmard JA, Mitchell JB, Thomas R, et al. Effects of repeated days of intensified training on muscle glycogen and swimming performance. Med Sci Sports Exerc. 1988 Jun;20(3):249-54.	226
Gleeson et al. (1999)	Gleeson M, McDonald WA, Pyne DB, Cripps AW, Francis JL, Fricker PA, et al. Salivary IgA levels and infection risk in elite swimmers. Med Sci Sports Exerc. 1999 Jan;31(1):67-73.	223
Morgan et al. (1988)	Morgan WP, Costill DL, Flynn MG, Raglin JS, O'Connor PJ. Mood disturbance following increased training in swimmers. Med Sci Sports Exerc. 1988 Aug;20(4):408-14.	213
**31. TABLE TENNIS**		
Rodrigues et al. (2002)	Rodrigues ST, Vickers JN, Williams AM. Head, eye and arm coordination in table tennis. J Sports Sci. 2002 Mar;20(3):187-200.	85
Raab et al. (2005)	Raab M, Masters RS, Maxwell JP. Improving the 'how' and 'what' decisions of elite table tennis players. Hum Mov Sci. 2005 Jun;24(3):326-44.	64
Kondric et al. (2013)	Kondric M, Zagatto AM, Sekulic D. The physiological demands of table tennis: a review. J Sports Sci Med. 2013;12(3):362-70.	44
Martinent and Ferrand (2009)	Martinent G, Ferrand C. A naturalistic study of the directional interpretation process of discrete emotions during high-stakes table tennis matches. J Sport Exerc Psychol. 2009 Jun;31(3):318-36.	42
Iino and Kojima (2009)	Iino Y, Kojima T. Kinematics of table tennis topspin forehands: effects of performance level and ball spin. J Sports Sci. 2009 Oct;27(12):1311-21.	40
**32. TAEKWONDO**		
Bridge et al. (2014)	Bridge CA, Ferreira da Silva Santos J, Chaabene H, Pieter W, Franchini E. Physical and physiological profiles of taekwondo athletes. Sports Med. 2014 Jun;44(6):713-33.	123
Campos et al. (2012)	Campos FA, Bertuzzi R, Dourado AC, Santos VG, Franchini E. Energy demands in taekwondo athletes during combat simulation. Eur J Appl Physiol. 2012 Apr;112(4):1221-8.	109
Matsushigue et al. (2009)	Matsushigue KA, Hartmann K, Franchini E. Taekwondo: Physiological responses and match analysis. J Strength Cond Res. 2009 Jul;23(4):1112-7.	106
Kazemi et al. (2006)	Kazemi M, Waalen J, Morgan C, White AR. A profile of olympic taekwondo competitors. J Sports Sci Med. 2006; 5(CSSI):114-21.	100
Falco et al. (2009)	Falco C, Alvarez O, Castillo I, Estevan I, Martos J, Mugarra F, et al. Influence of the distance in a roundhouse kick's execution time and impact force in Taekwondo. J Biomech. 2009 Feb 9;42(3):242-8.	95
**33. TENNIS**		
Nirschl (1992)	Nirschl RP. Elbow tendinosis/tennis elbow. Clin Sports Med. 1992 Oct;11(4):851-70.	295
Fernandez et al. (2006)	Fernandez J, Mendez-Villanueva A, Pluim BM. Intensity of tennis match play. Br J Sports Med. 2006 May;40(5):387-91; discussion 91.	201
Ellenbecker et al. (2002)	Ellenbecker TS, Roetert EP, Bailie DS, Davies GJ, Brown SW. Glenohumeral joint total rotation range of motion in elite tennis players and baseball pitchers. Med Sci Sports Exerc. 2002 Dec;34(12):2052-6.	188
O' Donoghue and Ingram (2001)	O' Donoghue P, Ingram B. A notational analysis of elite tennis strategy. J Sports Sci. 2001 Feb;19(2):107-15.	179
Mishra et al. (2014)	Mishra AK, Skrepnik NV, Edwards SG, Jones GL, Sampson S, Vermillion DA, et al. Efficacy of platelet-rich plasma for chronic tennis elbow: a double-blind, prospective, multicenter, randomized controlled trial of 230 patients. Am J Sports Med. 2014 Feb;42(2):463-71.	179
**34. TRAMPOLINE**		
Eliasson et al. (2002)	Eliasson K, Larsson T, Mattsson E. Prevalence of stress incontinence in nulliparous elite trampolinists. Scand J Med Sci Sports. 2002 Apr;12(2):106-10.	99
Nysted and Drogset (2006)	Nysted M, Drogset JO. Trampoline injuries. Br J Sports Med. 2006 Dec;40(12):984-7.	42
Da Roza et al. (2015)	Da Roza T, Brandao S, Mascarenhas T, Jorge RN, Duarte JA. Volume of training and the ranking level are associated with the leakage of urine in young female trampolinists. Clin J Sport Med. 2015 May;25(3):270-5.	34
Blajer and Czaplicki (2001)	Blajer W, Czaplicki A. Modeling and inverse simulation of somersaults on the trampoline. J Biomech. 2001 Dec;34(12):1619-29.	31
Hume et al. (1996)	Hume PA, Chalmers DJ, Wilson BD. Trampoline injury in New Zealand: emergency care. Br J Sports Med. 1996 Dec;30(4):327-30.	30
**35. TRIATHLON**		
Jeukendrup (2011)	Jeukendrup AE. Nutrition for endurance sports: marathon, triathlon, and road cycling. J Sports Sci. 2011;29 Suppl 1:S91-9.	168
Plews et al. (2012)	Plews DJ, Laursen PB, Kilding AE, Buchheit M. Heart rate variability in elite triathletes, is variation in variability the key to effective training? A case comparison. Eur J Appl Physiol. 2012 Nov;112(11):3729-41.	139
Coutts et al. (2007)	Coutts AJ, Wallace LK, Slattery KM. Monitoring changes in performance, physiology, biochemistry, and psychology during overreaching and recovery in triathletes. Int J Sports Med. 2007 Feb;28(2):125-34.	139
Millet et al. (2009)	Millet GP, Vleck VE, Bentley DJ. Physiological differences between cycling and running: lessons from triathletes. Sports Med. 2009;39(3):179-206.	128
Bentley et al. (2002)	Bentley DJ, Millet GP, Vleck VE, McNaughton LR. Specific aspects of contemporary triathlon: implications for physiological analysis and performance. Sports Med. 2002;32(6):345-59.	109
**36. VOLLEYBALL**		
Newton et al. (1999)	Newton RU, Kraemer WJ, Hakkinen K. Effects of ballistic training on preseason preparation of elite volleyball players. Med Sci Sports Exerc. 1999 Feb;31(2):323-30.	221
Ferretti et al. (1992)	Ferretti A, Papandrea P, Conteduca F, Mariani PP. Knee ligament injuries in volleyball players. Am J Sports Med. 1992 Mar-Apr;20(2):203-7.	208
Bahr and Bahr (1997)	Bahr R, Bahr IA. Incidence of acute volleyball injuries: a prospective cohort study of injury mechanisms and risk factors. Scand J Med Sci Sports. 1997 Jun;7(3):166-71.	206
Bahr et al. (1997)	Bahr R, Lian O, Bahr IA. A twofold reduction in the incidence of acute ankle sprains in volleyball after the introduction of an injury prevention program: a prospective cohort study. Scand J Med Sci Sports. 1997 Jun;7(3):172-7.	179
Verhagen et al. (2004)	Verhagen EA, Van der Beek AJ, Bouter LM, Bahr RM, Van Mechelen W. A one season prospective cohort study of volleyball injuries. Br J Sports Med. 2004 Aug;38(4):477-81.	178
**37. WALKING**		
Bassett et al. (2008)	Bassett DR, Jr., Pucher J, Buehler R, Thompson DL, Crouter SE. Walking, cycling, and obesity rates in Europe, North America, and Australia. J Phys Act Health. 2008 Nov;5(6):795-814.	362
Heller et al. (2001)	Heller MO, Bergmann G, Deuretzbacher G, Durselen L, Pohl M, Claes L, et al. Musculo-skeletal loading conditions at the hip during walking and stair climbing. J Biomech. 2001 Jul;34(7):883-93.	316
Ryan et al. (2006)	Ryan CG, Grant PM, Tigbe WW, Granat MH. The validity and reliability of a novel activity monitor as a measure of walking. Br J Sports Med. 2006 Sep;40(9):779-84.	272
Lee and Buchner (2008)	Lee IM, Buchner DM. The importance of walking to public health. Med Sci Sports Exerc. 2008 Jul;40(7 Suppl):S512-8.	254
Kelly et al. (2014)	Kelly P, Kahlmeier S, Gotschi T, Orsini N, Richards J, Roberts N, et al. Systematic review and meta-analysis of reduction in all-cause mortality from walking and cycling and shape of dose response relationship. Int J Behav Nutr Phys Act. 2014 Oct 24;11:132.	224
**38. WATERPOLO**		
Royal et al. (2006)	Royal KA, Farrow D, Mujika I, Halson SL, Pyne D, Abernethy B. The effects of fatigue on decision making and shooting skill performance in water polo players. J Sports Sci. 2006 Aug;24(8):807-15.	140
Smith (1998)	Smith HK. Applied physiology of water polo. Sports Med. 1998 Nov;26(5):317-34.	124
McMaster et al. (1991)	McMaster WC, Long SC, Caiozzo VJ. Isokinetic torque imbalances in the rotator cuff of the elite water polo player. Am J Sports Med. 1991 Jan-Feb;19(1):72-5.	107
Lupo et al. (2010)	Lupo C, Tessitore A, Minganti C, Capranica L. Notational analysis of elite and sub-elite water polo matches. J Strength Cond Res. 2010 Jan;24(1):223-9.	75
Tsekouras et al. (2005)	Tsekouras YE, Kavouras SA, Campagna A, Kotsis YP, Syntosi SS, Papazoglou K, et al. The anthropometrical and physiological characteristics of elite water polo players. Eur J Appl Physiol. 2005 Sep;95(1):35-41.	73
**39. WEIGHTLIFTING**		
Tricoli et al. (2005)	Tricoli V, Lamas L, Carnevale R, Ugrinowitsch C. Short-term effects on lower-body functional power development: weightlifting vs. vertical jump training programs. J Strength Cond Res. 2005 May;19(2):433-7.	169
Haff et al. (2005)	Haff GG, Carlock JM, Hartman MJ, Kilgore JL, Kawamori N, Jackson JR, et al. Force-time curve characteristics of dynamic and isometric muscle actions of elite women olympic weightlifters. J Strength Cond Res. 2005 Nov;19(4):741-8.	119
Hori et al. (2008)	Hori N, Newton RU, Andrews WA, Kawamori N, McGuigan MR, Nosaka K. Does performance of hang power clean differentiate performance of jumping, sprinting, and changing of direction? J Strength Cond Res. 2008 Mar;22(2):412-8.	115
Pearson et al. (2002)	Pearson SJ, Young A, Macaluso A, Devito G, Nimmo MA, Cobbold M, et al. Muscle function in elite master weightlifters. Med Sci Sports Exerc. 2002 Jul;34(7):1199-206.	113
Garhammer, 1980)	Garhammer J. Power production by Olympic weightlifters. Med Sci Sports Exerc. 1980 Spring;12(1):54-60.	111
**40. WRESTLING**		
Gould et al. (1993b)	Gould D, Eklund RC, Jackson SA. Coping strategies used by U.S. Olympic wrestlers. Res Q Exerc Sport. 1993 Mar;64(1):83-93.	203
Steen and Brownell (1990)	Steen SN, Brownell KD. Patterns of weight loss and regain in wrestlers: has the tradition changed? Med Sci Sports Exerc. 1990 Dec;22(6):762-8.	176
Kraemer et al. (2001)	Kraemer WJ, Fry AC, Rubin MR, Triplett-McBride T, Gordon SE, Koziris LP, et al. Physiological and performance responses to tournament wrestling. Med Sci Sports Exerc. 2001 Aug;33(8):1367-78.	168
Oppliger et al. (1996)	Oppliger RA, Case HS, Horswill CA, Landry GL, Shelter AC. American College of Sports Medicine position stand. Weight loss in wrestlers. Med Sci Sports Exerc. 1996 Jun;28(6):ix-xii.	141
Webster et al. (1990)	Webster S, Rutt R, Weltman A. Physiological effects of a weight loss regimen practiced by college wrestlers. Med Sci Sports Exerc. 1990 Apr;22(2):229-34.	135

**Table 5:** Top-5 articles on winter sports.

**References**	**Articles**	**Number citations**
**1. ALPINE SKIING**		
Ettlinger et al. (1995)	Ettlinger CF, Johnson RJ, Shealy JE. A method to help reduce the risk of serious knee sprains incurred in alpine skiing. Am J Sports Med. 1995 Sep-Oct;23(5):531-7.	173
Florenes et al. (2009)	Florenes TW, Bere T, Nordsletten L, Heir S, Bahr R. Injuries among male and female World Cup alpine skiers. Br J Sports Med. 2009 Dec;43(13):973-8.	104
Burtscher et al. (2008)	Burtscher M, Gatterer H, Flatz M, Sommersacher R, Woldrich T, Ruedl G, et al. Effects of modern ski equipment on the overall injury rate and the pattern of injury location in Alpine skiing. Clin J Sport Med. 2008 Jul;18(4):355-7.	95
Bere et al. (2011)	Bere T, Florenes TW, Krosshaug T, Koga H, Nordsletten L, Irving C, et al. Mechanisms of anterior cruciate ligament injury in World Cup alpine skiing: a systematic video analysis of 20 cases. Am J Sports Med. 2011 Jul;39(7):1421-9.	90
Pujol et al. (2007)	Pujol N, Blanchi MP, Chambat P. The incidence of anterior cruciate ligament injuries among competitive Alpine skiers: a 25-year investigation. Am J Sports Med. 2007 Jul;35(7):1070-4.	89
**2. BIATHLON**		
Vickers and Williams (2007)	1. Vickers JN, Williams AM. Performing under pressure: the effects of physiological arousal, cognitive anxiety, and gaze control in biathlon. J Mot Behav. 2007 Sep;39(5):381-94.	160
Heinicke et al. (2005)	1. Heinicke K, Heinicke I, Schmidt W, Wolfarth B. A three-week traditional altitude training increases hemoglobin mass and red cell volume in elite biathlon athletes. Int J Sports Med. 2005 Jun;26(5):350-5.	81
Hoffman et al. (1992)	1. Hoffman MD, Gilson PM, Westenburg TM, Spencer WA. Biathlon shooting performance after exercise of different intensities. Int J Sports Med. 1992 Apr;13(3):270-3.	64
Rundell and Bacharach (1995)	1. Rundell KW, Bacharach DW. Physiological characteristics and performance of top U.S. biathletes. Med Sci Sports Exerc. 1995 Sep;27(9):1302-10.	38
Rundell (1995)	1. Rundell KW. Treadmill roller ski test predicts biathlon roller ski race results of elite U.S. biathlon women. Med Sci Sports Exerc. 1995 Dec;27(12):1677-85.	35
**3. BOBSLEIGH**		
Dabnichki and Avital (2006)	Dabnichki P, Avital E. Influence of the postion of crew members on aerodynamics performance of two-man bobsleigh. J Biomech. 2006;39(15):2733-42.	29
Haralambie et al. (1976)	Haralambie G, Cerny FJ, Huber G. Serum enzyme levels after bobsled racing. J Sports Med Phys Fitness. 1976 Mar;16(1):54-6.	11
Reid (2003)	Reid SA. Stress fracture of the ulna in an elite bobsled brakeman. Clin J Sport Med. 2003 Sep;13(5):306-8.	4
Lopes and Alouche (2016)	Lopes AD, Alouche SR. Two-Man Bobsled Push Start Analysis. J Hum Kinet. 2016 Apr 1;50:63-70.	4
Okada et al. (1972)	Okada A, Miyake H, Takizawa A, Minami M. A study on the excreted catecholamines in the urine of Bobsleigh-tobogganing contestants. J Sports Med Phys Fitness. 1972 Jun;12(2):71-5.	3
**4. CURLING**		
Bradley (2009)	Bradley JL. The sports science of curling: a practical review. J Sports Sci Med. 2009;8(4):495-500.	13
Robertson et al. (2017)	Reeser JC, Berg RL. Self-reported injury patterns among competitive curlers in the United States: a preliminary investigation into the epidemiology of curling injuries. Br J Sports Med. 2004 Oct;38(5):E29.	5
Berry et al. (2013)	Berry JW, Romanick MA, Koerber SM. Injury type and incidence among elite level curlers during world championship competition. Res Sports Med. 2013;21(2):159-63.	4
Stone et al. (2018)	Stone RC, Rakhamilova Z, Gage WH, Baker J. Curling for Confidence: Psychophysical Benefits of Curling for Older Adults. J Aging Phys Act. 2018 Apr 1;26(2):267-75.	2
Pojskic et al. (2020)	Pojskic H, McGawley K, Gustafsson A, Behm DG. The Reliability and Validity of a Novel Sport-Specific Balance Test to Differentiate Performance Levels in Elite Curling Players. J Sports Sci Med. 2020 Jun;19(2):337-46.	1
**5. ICE HOCKEY**		
Philippon et al. (2010)	Philippon MJ, Weiss DR, Kuppersmith DA, Briggs KK, Hay CJ. Arthroscopic labral repair and treatment of femoroacetabular impingement in professional hockey players. Am J Sports Med. 2010 Jan;38(1):99-104.	207
Tyler et al. (2001)	Tyler TF, Nicholas SJ, Campbell RJ, McHugh MP. The association of hip strength and flexibility with the incidence of adductor muscle strains in professional ice hockey players. Am J Sports Med. 2001 Mar-Apr;29(2):124-8.	205
Sherar et al. (2007)	Sherar LB, Baxter-Jones AD, Faulkner RA, Russell KW. Do physical maturity and birth date predict talent in male youth ice hockey players? J Sports Sci. 2007 Jun;25(8):879-86.	195
Williamson and Goodman (2006)	Williamson IJ, Goodman D. Converging evidence for the under-reporting of concussions in youth ice hockey. Br J Sports Med. 2006 Feb;40(2):128-32; discussion−32.	189
Flik et al. (2005)	Flik K, Lyman S, Marx RG. American collegiate men's ice hockey: an analysis of injuries. Am J Sports Med. 2005 Feb;33(2):183-7.	165
**6. LUGE**		
Platzer et al. (2009)	Platzer HP, Raschner C, Patterson C. Performance-determining physiological factors in the luge start. J Sports Sci. 2009 Feb 1;27(3):221-6.	20
Cummings et al. (1997)	Cummings RS, Jr., Shurland AT, Prodoehl JA, Moody K, Sherk HH. Injuries in the sport of luge. Epidemiology and analysis. Am J Sports Med. 1997 Jul-Aug;25(4):508-13.	17
Crossland et al. (2011)	Crossland BW, Hartman JE, Kilgore JL, Hartman MJ, Kaus JM. Upper-body anthropometric and strength measures and their relationship to start time in elite luge athletes. J Strength Cond Res. 2011 Oct;25(10):2639-44.	10
Mossner et al. (2011)	Mossner M, Hasler M, Schindelwig K, Kaps P, Nachbauer W. An approximate simulation model for initial luge track design. J Biomech. 2011 Mar 15;44(5):892-6.	6
Lembert et al. (2011)	Lembert S, Schachner O, Raschner C. Development of a measurement and feedback training tool for the arm strokes of high-performance luge athletes. J Sports Sci. 2011 Dec;29(15):1593-601.	4
**7. NORDIC SKIING**		
Millet and Lepers (2004)	Millet GY, Lepers R. Alterations of neuromuscular function after prolonged running, cycling and skiing exercises. Sports Med. 2004;34(2):105-16.	239
Holmberg et al. (2005)	Holmberg HC, Lindinger S, Stoggl T, Eitzlmair E, Muller E. Biomechanical analysis of double poling in elite cross-country skiers. Med Sci Sports Exerc. 2005 May;37(5):807-18.	177
Hoff et al. (1999)	Hoff J, Helgerud J, Wisloff U. Maximal strength training improves work economy in trained female cross-country skiers. Med Sci Sports Exerc. 1999 Jun;31(6):870-7.	121
Grimsmo et al. (2010)	Grimsmo J, Grundvold I, Maehlum S, Arnesen H. High prevalence of atrial fibrillation in long-term endurance cross-country skiers: echocardiographic findings and possible predictors–a 28-30 years follow-up study. Eur J Cardiovasc Prev Rehabil. 2010 Feb;17(1):100-5.	118
Andersson et al. (2010)	Andersson E, Supej M, Sandbakk O, Sperlich B, Stoggl T, Holmberg HC. Analysis of sprint cross-country skiing using a differential global navigation satellite system. Eur J Appl Physiol. 2010 Oct;110(3):585-95.	87
**8. SKATING**		
Gould et al. (1993a)	Gould D, Finch LM, Jackson SA. Coping strategies used by national champion figure skaters. Res Q Exerc Sport. 1993 Dec;64(4):453-68.	142
Herzog et al. (1991)	Herzog W, Guimaraes AC, Anton MG, Carter-Erdman KA. Moment-length relations of rectus femoris muscles of speed skaters/cyclists and runners. Med Sci Sports Exerc. 1991 Nov;23(11):1289-96.	121
van Ingen Schenau et al. (1994)	van Ingen Schenau GJ, de Koning JJ, de Groot G. Optimisation of sprinting performance in running, cycling and speed skating. Sports Med. 1994 Apr;17(4):259-75.	90
van Ingen Schenau (1982)	van Ingen Schenau GJ. The influence of air friction in speed skating. J Biomech. 1982;15(6):449-58.	90
Foster et al. (1999)	Foster C, Rundell KW, Snyder AC, Stray-Gundersen J, Kemkers G, Thometz N, et al. Evidence for restricted muscle blood flow during speed skating. Med Sci Sports Exerc. 1999 Oct;31(10):1433-40.	70
**9. SKELETON**		
Bullock et al. (2008)	Bullock N, Martin DT, Ross A, Rosemond CD, Jordan MJ, Marino FE. Acute effect of whole-body vibration on sprint and jumping performance in elite skeleton athletes. J Strength Cond Res. 2008 Jul;22(4):1371-4.	55
Bullock et al. (2009)	Bullock N, Gulbin JP, Martin DT, Ross A, Holland T, Marino F. Talent identification and deliberate programming in skeleton: ice novice to Winter Olympian in 14 months. J Sports Sci. 2009 Feb 15;27(4):397-404.	51
Sands et al. (2005)	Sands WA, Smith LS, Kivi DM, McNeal JR, Dorman JC, Stone MH, et al. Anthropometric and physical abilities profiles: US National Skeleton Team. Sports Biomech. 2005 Jul;4(2):197-214.	31
Zanoletti et al. (2006)	Zanoletti C, La Torre A, Merati G, Rampinini E, Impellizzeri FM. Relationship between push phase and final race time in skeleton performance. J Strength Cond Res. 2006 Aug;20(3):579-83.	26
Bullock et al. (2007)	Bullock N, Martin DT, Ross A, Rosemond D, Marino FE. Effect of long haul travel on maximal sprint performance and diurnal variations in elite skeleton athletes. Br J Sports Med. 2007 Sep;41(9):569-73; discussion 73.	25
**10. SNOWBOARD**		
Bladin et al. (1993)	Bladin C, Giddings P, Robinson M. Australian snowboard injury data base study. A four-year prospective study. Am J Sports Med. 1993 Sep-Oct;21(5):701-4.	109
Kim et al. (2012)	Kim S, Endres NK, Johnson RJ, Ettlinger CF, Shealy JE. Snowboarding injuries: trends over time and comparisons with alpine skiing injuries. Am J Sports Med. 2012 Apr;40(4):770-6.	86
Pino and Colville (1989)	Pino EC, Colville MR. Snowboard injuries. Am J Sports Med. 1989 Nov-Dec;17(6):778-81.	85
Tarazi et al. (1999)	Tarazi F, Dvorak MF, Wing PC. Spinal injuries in skiers and snowboarders. Am J Sports Med. 1999 Mar-Apr;27(2):177-80.	83
Ronning et al. (2001)	Ronning R, Ronning I, Gerner T, Engebretsen L. The efficacy of wrist protectors in preventing snowboarding injuries. Am J Sports Med. 2001 Sep-Oct;29(5):581-5.	77

## Results

The bibliometric analysis was performed on 50 Olympic sports or disciplines in 116 “sport sciences” journals and led to the selection of 25,003 articles with a total number of ~600,000 citations.

**Figure 3:**
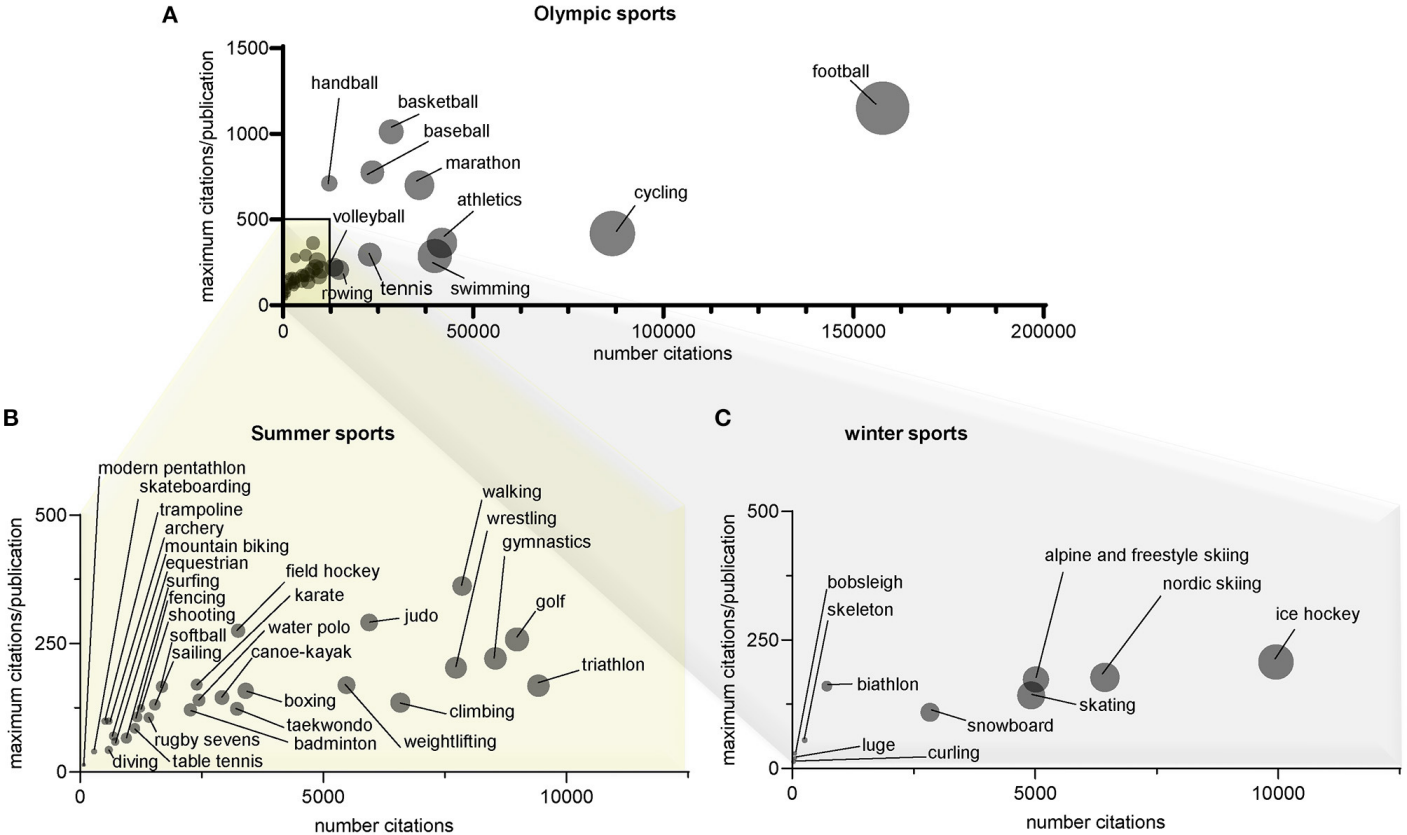
Publication and citation numbers across all Olympic sports. All Olympic sports are depicted in panel **(A)**. Zooms of sports with citation ranges from 0 to 12,500 citations and maximum citations/publication of 500 are provided in **(B)** for summer and **(C)** for winter Olympic sports. Bubble sizes reflect numbers of publications for each sport relative to the greatest bubble of each panel. Highest publication numbers: **(A)** football—4,937, **(B)** golf—491, and **(C)** ice-hockey—540.

There is a large range of articles and citations across sports (Figure 3). Nine sports (football, cycling, athletics, swimming, distance & marathon running, basketball, baseball, tennis, and rowing) were involved in 69% of the articles and 75% of the citations. Football (soccer) was the most cited sport, with 19.7 and 26.3% of the total numbers of articles and citations, respectively. Scientific research has been published on all sports, but 11 sports (biathlon, mountain biking, archery, diving, trampoline, skateboarding, skeleton, modern pentathlon, luge, bobsleigh, and curling) accumulated a total of fewer than 50 publications. While ice hockey is the most prominently represented winter sport in the scientific literature, winter sports overall have produced minor scientific output.

**Figure 4:**
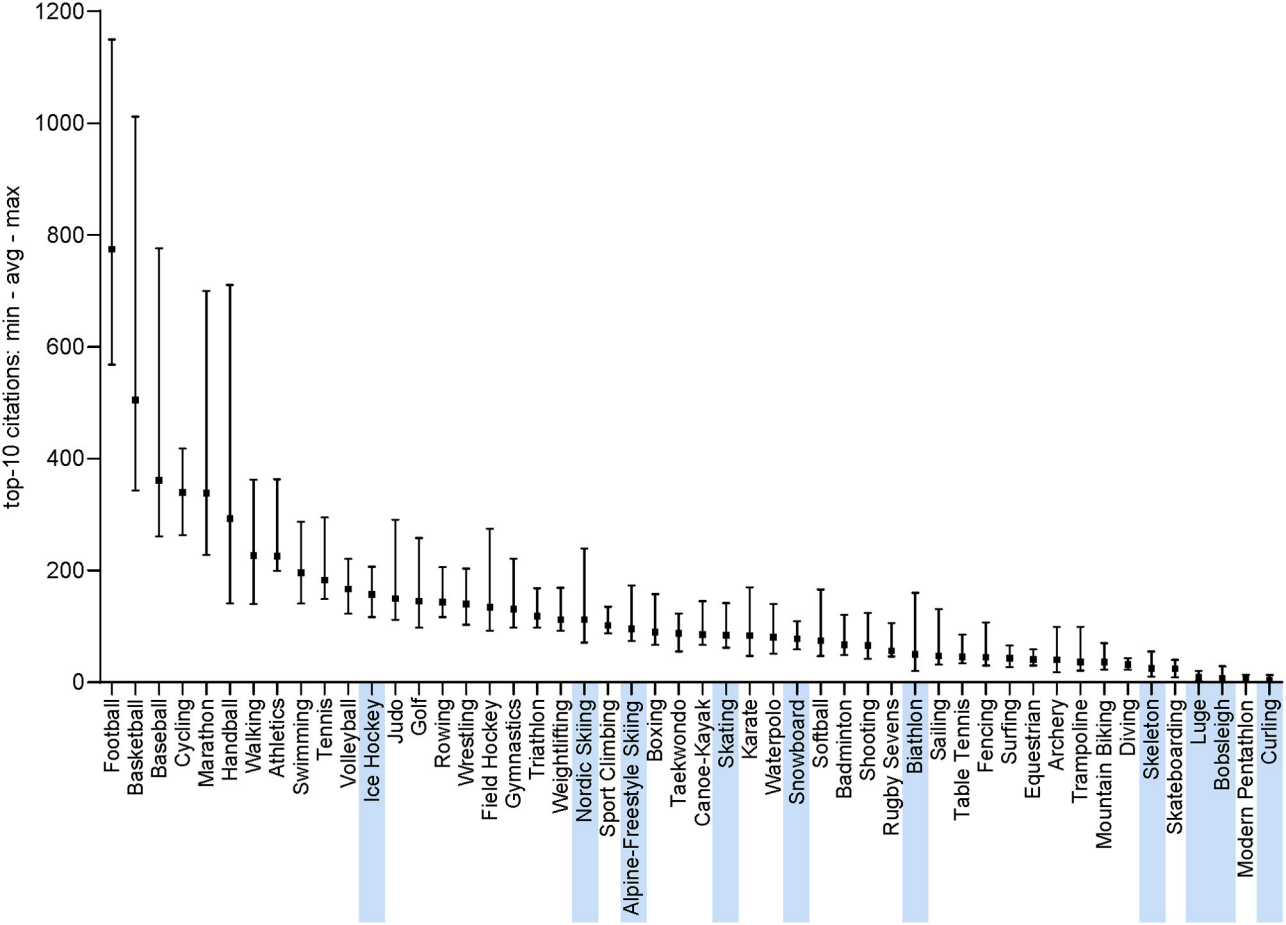
Maximum (upper boundary), average (square) and minimum (lower boundary) numbers of citations of the top-10 publications of each sport. Winter sports are highlighted.

The analysis of the level and depth of the 10 most cited articles in every sports confirms this discrepancy across sports (Figure 4). This analysis confirms the results in terms of total publications across sports (Figure 3). Some sports (e.g., basketball and baseball) have highly cited articles (i.e., based on the average number of citations of the 10 most cited articles). This is also the case for handball, which has a relatively low number of citations (Figure 3) but a few highly cited articles (Figure 4).

**Figure 5:**
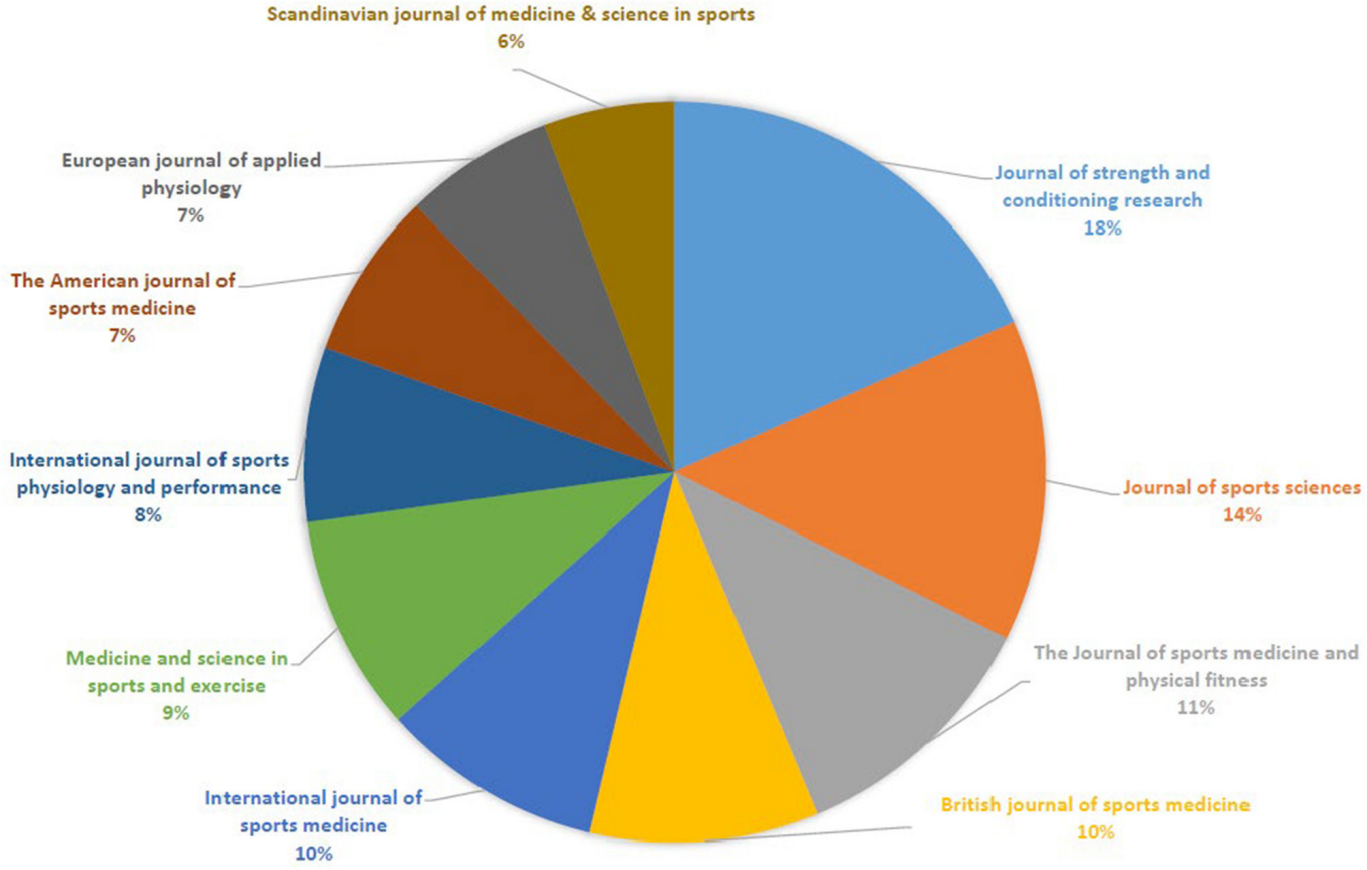
Distribution of major “Olympic sports science” publications across journals for all Olympic sports.

Next, we analyzed the distribution of “Olympic sport sciences” publications across journals. This investigation revealed that only a small number of journals have published the greatest part of such articles. Merely six journals (*J Strength Cond Res*, 10.0%; *J Sports Sci*, 7.7%; *J Sports Med Phys Fitness*, 6.2%; *Br J Sports Med*, 5.5%; *Int J Sports Med*, 5.3%; and *Med Sci Sports Exerc*, 5.2%) of the 116 included in our search had published 40% of all publications (Figure 5). Some factors (including the nature of the sport as well as geographical and cultural factors and the composition of the editorial board), however, seem to have influenced the ratio of articles on specific sports appearing in different journals. For example, baseball articles have been published mainly in orthopedic or “sports medicine” journals (1. *Am J Sports M*ed; 2. *J Shoulder Elbow Surgery*, and 3. *Orthop J Sports Med*) while basketball articles were published in conditioning or “sport sciences” journals (1. *J Strength Cond Res*; 2. *J Sports Sci*, and 3. *J Sports Med Phys Fitness*). Tennis articles are overrepresented in *Br J Sports Med*, and Nordic skiing articles in *Scand J Med Sci Sports*.

**Figure 6:**
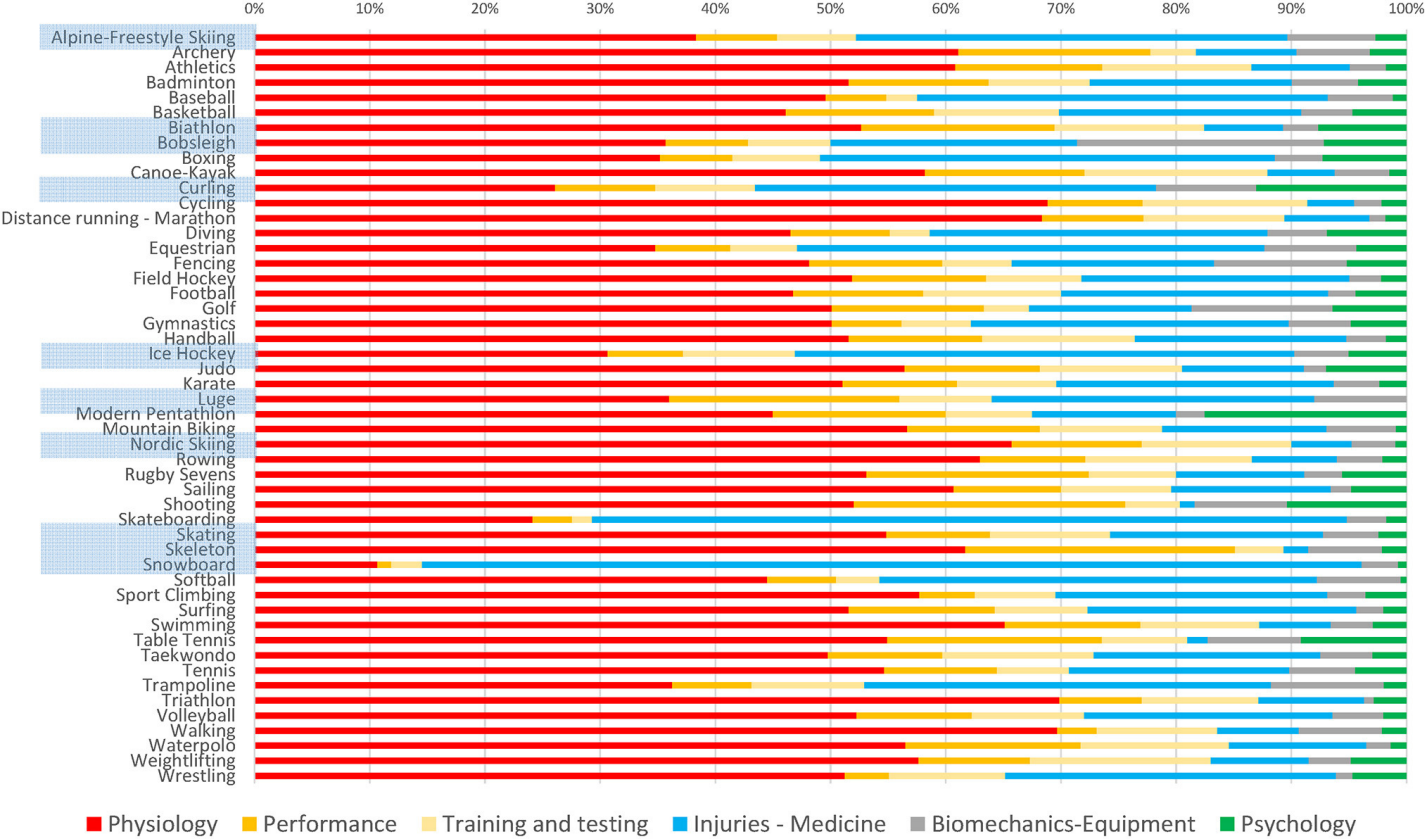
Distribution of publications between six research topics across all summer and winter Olympic sports. Winter sports are highlighted.

Finally, the distribution of different research topics (i.e., physiology, performance, training and testing, injuries and medicine, biomechanics, and psychology) varies largely among sports (Figure 6).

## Discussion

The present bibliometric analysis is the first to quantify the bibliometric across all summer and winter Olympic sports. This comprehensive review provides interesting outcomes that are summarized briefly here and discussed afterwards:

1. There is a large difference in scientific output among sports, with nine sports representing 75% of the citations and 11 having a total of fewer than 50 associated publications.
2. Football (soccer) is by far the leading Olympic sport in terms of bibliometrics.
3. Team sports, particularly American professional sports (i.e., baseball, basketball, ice hockey), generate high scientific interest.
4. Overall, winter sports generate minor scientific output.
5. Most articles have been published in a limited number of journals.
6. Whether the inclusion of a sport in the Olympic programme translates into an increase in scientific publications remains unclear.
7. We also report some influence of local/cultural factors and/or of editorial board composition on the importance of a given sport in a given journal.
8. Finally, the distribution of articles among six main research topics (i.e., physiology, performance, training and testing, injuries and medicine, biomechanics, and psychology) highlights the (scientific) performance determinants of each sport.

### Large Differences Between Sports

To our knowledge, there has been no comprehensive analysis and comparison of the largely different physical demands across all Olympic sports since the multifactorial determinants of performance within and across all Olympic sports render such analysis difficult. For example, curling and shooting have little in common with boxing, triathlon, or freestyle skiing. A quantitative comparison of the “sport sciences” literature across all these sports, on the other hand, is feasible and provides information on the scientific importance of the various sports.

Our analysis revealed that only nine sports (football, cycling, athletics, swimming, distance & marathon running, basketball, baseball, tennis, and rowing) represented 69% of the articles and 75% of the citations, while 11 sports (biathlon, mountain biking, archery, diving, trampoline, skateboarding, skeleton, modern pentathlon, luge, bobsleigh, and curling) accumulated a total of fewer than 50 publications.

Why a given sport attracts many publications certainly depends on a number of variables. Unsurprisingly, the sports with the most published and cited articles are very popular, and most of them are long established in the Olympic programme, e.g., from the start in 1896–1900, with the exceptions of basketball (1936) and baseball (1992). While the time since inclusion in the Olympic programme seems to be a key criterion for the attraction of scientific interest for some sports, this appears not to be the case for other, even “traditional” Olympic sports, such as wrestling or fencing (both Olympic sports since 1896). Another criterion for scientific attractiveness may be individual vs. team sports. Team sports are highly investigated, as is confirmed by our finding that five team sports (football, basketball, volleyball, handball, and ice hockey) rank among the top 12 most cited of the 50 sports analyzed. Conversely, the impact on the scientific literature is lower in other team sports, including field hockey, water polo and rugby sevens (a recent inclusion in the Olympic programme).

When analyzing the individual sports, it is noteworthy that the sports in which performance is determined mainly by energy (aerobic and anaerobic) production—as conceptually opposed to “motor control” or “technical” sports categories—have led to a larger scientific output. Sports belonging to the first category include cycling, athletics, swimming, distance running—marathon and rowing, all of which rank among the top 10 most cited sports. Baseball (see below) and tennis are the exceptions, representing technical sports in this top 10 ranking. Supporting this notion, the technical sports golf (despite its media prominence) and gymnastics (one of the most important Olympic sports) are less frequently cited than, for example, triathlon. One may speculate that more energy-reliant sports may benefit to a greater extent from general scientific support/knowledge (i.e., exercise physiology) than the more “technical” sports (i.e., motor control). This suggestion is corroborated by the importance of the “physiology” research topic (see chapter 8 and Figure 6) across most sports. However, the limitation of our search to PubMed and the biomedical literature may partially account for this result.

It is very challenging to clearly appreciate why a sport attracts the interest of sport scientists. We do not exclude the possibility that this effect can be explained by more general factors (e.g., a general increase in publication numbers in recent decades). Olympic sports may be of higher scientific interest to sport scientists than non-Olympic sports. This may be related to a trend of scientific support increasingly becoming a key component of elite performance. Many scientists of excellent scientific/academic background (i.e., Dupont et al., 2005; Bangsbo et al., 2008 in football, Mountjoy et al., 2016; Mujika et al., 2019 in swimming, Mujika et al., 2019 in athletics, Jones et al., 2021 in distance running, and Hebert-Losier et al., 2017; Solli et al., 2017 in Nordic skiing, to name only a few—we apologize to many other colleagues who deserve to be on this list) are indeed servicing and advising elite athletes or teams while in parallel producing outstanding scientific research that is sometimes relevant for coaches. Until recently, the translation of “sport sciences” research to practice was often poor (Bishop, 2008), and interdependence between the practical and scientific impacts of “sport sciences” research has frequently been advocated (Coutts, 2016; Brocherie and Beard, 2020). Elite sports organizations require embedded, fast-moving, service-providing applied research scientists as well as slow-thinking researchers (Sandbakk, 2018), who, working together, will carry on producing sport-specific research.

Most elite sports institutes (e.g., Insep in France https://labos-recherche.insep.fr/fr), the IOC (https://olympics.com/ioc/medical-and-scientific-commission) and some National Olympic Committees and national and international governing bodies (e.g., World Athletics https://www.worldathletics.org/about-iaaf/health-science) have developed scientific committees to stimulate research on specific topics according to their needs. Examples are programmes with the aim of implementing new rules for the protection of athletes' health by limiting concussion (Stokes et al., 2021) or heat stress (Mountjoy et al., 2012). Although the scientific support and service sector has grown tremendously in the last two decades, the impact of scientific support on sports performance remains difficult to quantify.

However, while we believe that sport-specific attractiveness is due mainly to the importance of the sport itself, it is beyond the scope of the present review to relate the present bibliometric information to other sport characteristics, such as but not limited to the number of participants, economic weight and media exposure. These points are briefly discussed in the present review but certainly also contribute to the importance of a particular sport in the scientific literature. The quality of servicing scientists at the club, federation, or sport institute levels may be a factor of influence, but the vast majority of these sports publications seem to have come from academic (i.e., employed by universities or research organizations) researchers. With the evolution of the performance support model within the professional and elite sporting environment, deemed necessary to integrate an applied research process to bridge the gap between scientists and practitioners (Brocherie and Beard, 2020), the scientific publication landscape may change in the future, even for less attractive sports.

### Football (Soccer) Dominates the Scientific Literature

The dominance of football in the “sport sciences” literature is impressive. Football represents 19.7 and 26.3% of the total number of articles and citations, respectively (Figure 3), despite its relatively low importance with regard to Olympic medal counts (i.e., 2 of 339 gold medals at Tokyo 2020 vs. 48 in athletics, 37 in swimming and 12 in Nordic skiing or skating at Beijing 2022, to cite only the main Olympic sports). The reasons, therefore, are unrelated to the Olympics and likely are attributable to its general popularity and associated economic characteristics. Football is the most popular sport worldwide (e.g., the global audience at the FIFA World Cup 2018 was estimated to be 3.57 billion people). Half of the total revenue of the sports industry is gained by competitive sports of the spectator sports sector, amounting to approximately US$250 billion in turnover each year. The share of football accounts for an estimated 43% of this revenue and thus is much larger than the shares of other Olympic sports or even of other US professional sports; it is almost equal to the combined revenue from all US sports, including American football (13%), baseball (12%), Formula 1 auto racing (7%), basketball (6%), ice hockey (4%) and tennis (4%) (https://www.researchandmarkets.com/reports/5022446/sports-global-market-report-2020-30-covid-19). While our findings are in line with previous results (Brito et al., 2018), the consequences and implications of the scientific dominance of football remain unclear. It is tempting to relate such scientific proliferation to the already well-organized performance support services within professional and elite football (Brocherie and Beard, 2020). However, to our knowledge, there has been no comprehensive analysis of the number of scientists working in professional football, even if it is obvious that this segment has grown considerably in the last decade, especially in the clubs of the five major football leagues in Europe (i.e., England, Spain, Germany, Italy, and France). This may have provided an edge over many other sports that are still in the process of establishing efficient structures (e.g., some leading US sports league franchises) (Brocherie and Beard, 2020).

### Importance of Team Sports, Particularly American Professional Sports

Several North American professional sports are highly ranked in terms of bibliometrics. As for football, it is likely that the economic characteristics of the main North American national leagues (Major League Baseball, National Basketball Association, and National Hockey League (estimated at 5.5, 4.6, and 2.2 billion US dollars in 2015, respectively; https://www.ameriresearch.com/global-football-sports-market/) may be one reason for the scientific interest in these sports. Moreover, “sport sciences” is a well-established academic discipline, and the USA is a leading contributor in this field, as is exemplified by the largest “sport sciences” society worldwide, *American College of Sports Medicine* (ACSM) (www.acsm.org), with more than 50,000 members and certified professionals from 90 countries around the globe.

In line with other team sports (e.g., volleyball, handball, and field hockey), publications related to injuries (prevention and rehabilitation) are relatively more important in team sports (>20% of the total sport-specific articles; Figure 6) than in the main individual sports (cycling, athletics, swimming, distance running—marathon, etc.). This may stem from a higher degree of professionalization and therefore specialization of permanent full-time medical staff in team sports due to the economic power of these sports and the financial value of professional players.

### Winter Sports Generate Minor Scientific Production

Despite some parts of the world being particularly passionate about winter sports (e.g., Sweden and Norway for Nordic skiing, Russia and Canada for ice hockey, and Austria and Switzerland for alpine skiing), the audience for winter sports and number of participants remain comparatively low worldwide. This is likely due primarily to geographical and climatic limitations (i.e., especially the lack of snow) for the development of winter sports. The lower importance of winter sports becomes clear when comparing the latest summer and winter Olympic games. A record number of 2,922 athletes from 92 countries participated in the Pyeongchang 2018 Winter Games, while 11,362 athletes from 204 countries participated in the Rio de Janeiro 2016 Summer Games. A similar discrepancy is observed with regard to the number of sports and disciplines, with 102 events in 7 sports (and 15 disciplines) at the 2018 winter Olympic games vs. 306 events in 28 sports and 43 disciplines at the 2016 summer games.

In the European Nordic countries, sport sciences have a long tradition of excellence, owing primarily to the work of famous pioneers in exercise physiology (e.g., Saltin and Astrand, 1967) who performed early studies, including some on Nordic skiers. This might partly explain why Nordic skiing is the second most cited winter sport (after ice hockey—see above).

### Most Articles are Published in a Limited Number of Journals

Six journals of 116 included in our search (*J Strength Cond Res*, 10.0%; *J Sports Sci*, 7.7%; *J Sports Med Phys Fitness*, 6.2%; *Br J Sports Med*, 5.5%; *Int J Sports Med*, 5.3%; and *Med Sci Sports Exerc*, 5.2%) contained 40% of all analyzed publications. These leading journals publish articles predominantly on applied research as well as on conditioning or training and testing (e.g., *J Strength Cond Res, J Sports Med Phys Fitness*, and *J Sports Sci)*. Some are tightly connected to powerful organizations (e.g., *Br J Sports Med*, which regularly publishes reports or statements of the IOC, or *Med Sci Sports Exerc*, which belongs to the ACSM).

Our search included 116 journals, but many of them do not publish “biomedical” articles (accessible in PubMed) specific to any of the Olympic sports. The scope of some journals is very broad (e.g., applied physiology in *J Appl Physiol*) or very narrow (e.g., *High Alt Med Biol*); articles focusing on one given sport in those journals are thus less frequent. Many journals are furthermore relatively new in PubMed (e.g., *Int J Sports Physiol Perf* and *Front Sports Active Living*). Finally, the fact that most articles are published in only a few journals may render questionable the profusion of (too?) many journals in the “sport sciences” field, which has been growing since the early 2000's.

### The Entry of a Sport Into the Olympic Programme Translates Into an Increase in Scientific Publications

We scrutinized whether the Olympic entrance of the “recent” Olympic sports (e.g., inserted in the Olympic programme in the last 25 years: snowboard in 1998; trampoline, triathlon, and taekwondo in 2000; rugby sevens in 2016; and surfing, karate, sport climbing, and skateboarding in 2020), might have impacted their specific scientific attractiveness. Figure 2 shows the evolution of yearly citation numbers between 1990 and 2020 with the date of the entrance into the Olympic programme for four “recent” sports (snowboard, triathlon taekwondo, and rugby sevens). Whether entrance into the programme has a positive effect remains unclear, even if an increase in the publication rate is observable 6–8 years after (for snowboard and taekwondo) or several years before (as is clearly shown for rugby sevens and triathlon) nomination as an Olympic sport. Overall, the “Olympic legacy” does not seem to stimulate a large increase in the volume of articles or citations (Thomas et al., 2016).

Of these “recent” Olympic sports, triathlon is by far the most productive of scientific output (Figure 2). As discussed in chapter 1, this may stem from the nature of the sport, which is highly energetic and of interest to physiologists, while other “recent” sports are less aerobic.

### Local/Cultural Influence and/or Influence of Editorial Board Composition

Sports carry strong cultural and political meanings for their practitioners and spectators and powerfully symbolize identities and communities (Millet and Giulianotti, 2019). It is therefore not surprising—and in a sense reassuring in our globalized world—to find that a local sporting culture can impact the scientific output, as is testified by the overrepresentation of alpine and Nordic skiing in *Scand J Med Sci Sport*. “Sport sciences” (like most other scientific fields) are dominated by Anglo-Saxon countries (especially the USA, UK, Australia, and Canada). As has recently been observed (Pyne, 2021), research in several of the world's leading sporting nations (e.g., Russia, China, Japan, and South Korea; all top 8 nations at the 2016 Summer Olympic Games) is underrepresented in “sport sciences” journals that are published mostly in English. It is beyond the scope of this review to analyze all the other potential factors or barriers (economic, political, religious, gender based, etc.) that bias the over- vs. under-representation of a given sport in the “sport sciences” literature, but more cultural, geographical and gender diversity is needed. Another observation is the influence of the composition of the editorial boards of the journals on editorial policy as well as the published content. All the above-mentioned factors influence regular publications on certain sports in journals, such as rugby sevens in *Int J Sports Physiol Perf or* tennis in *Br J Sports Med*, while some sports that are extremely popular in Asia (taekwondo and table tennis) lack comparable platforms for scientific exchange.

### Relative Distribution of Six Main Research Topics Across Sports

We analyzed the relative distribution of six research topics (i.e., physiology, performance, training and testing, injuries and medicine, biomechanics, and psychology) across all summer and winter Olympic sports publications since the analysis may provide informational particularities that are especially relevant for research on these sports or on the determinants of performance, which vary considerably among sports. For example, it has long been known that maximal aerobic power is paramount in cross-country skiing, cycling, distance running and rowing, as is evidenced by the high maximal oxygen consumption (VO_2max_) values in top performers in these sports (Haugen et al., 2018), who reach VO_2max_ values of >90 ml/kg/min (Millet and Jornet, 2019). Although “physiology” covers other aspects than aerobic capacity, many publications (approximately two-thirds) on sports such as triathlon, swimming, and walking concern physiological aspects due to these sports' high reliance on aerobic capacities.

Whereas, the scientific literature on many sports is dominated by physiological topics, research on other sports focuses on associated injuries-illnesses. The topic “injuries and medicine” is paramount (i.e., > 40% of related publications) in five summer (baseball, boxing, equestrian, skateboarding, and softball) and 4 winter (alpine freestyle skiing, curling, ice hockey, and snowboarding) sports. Of the publications, 65% of those on skateboarding and 82% of those on snowboarding concern injuries. Deeper analyses of these publications are required to differentiate the types and causes of injuries between contact sports (e.g., boxing and ice hockey), sports inducing falls (equestrian, alpine skiing, snowboarding, and skateboarding), and sports inducing overuse injuries (e.g., elbow injury in baseball and softball). The “injuries and illnesses prevention and incidence” topic is of the highest priority in elite sports; the IOC medical and scientific commission (https://olympics.com/ioc/medical-and-scientific-commission) publishes regular reports on injuries and illness incidences in the summer (Soligard et al., 2017) and winter (Soligard et al., 2019) Olympic games. During the last summer games in Rio de Janeiro in 2016, the injury incidence ranged from 38% in BMX cycling to 0–3% in canoeing, rowing, shooting, archery, swimming, golf, and table tennis, while the illness incidence was 10–12% in diving, swimming, sailing, canoeing-kayaking and equestrian (Soligard et al., 2017). During the last winter games in Pyeongchang in 2018, the injury incidence was highest (20–28%) in freestyle skiing and snowboarding and lowest (2–6%) in Nordic combined, biathlon, snowboard slalom, moguls, and cross-country skiing. The illness incidences ranged between 13 and 15% in biathlon, curling, bobsleigh, and snowboard slalom (Soligard et al., 2019).

Surprisingly, in every sport, the number of publications on psychology-related topics is quite low. Only for curling, shooting, and modern pentathlon are >10% of the sport-specific publications related to psychology, followed by table tennis. All these sports require extreme accuracy and self-control. The possibility that this low representation of psychological articles relates to the applied methodology (e.g., the database searched was PubMed) cannot be excluded, but most of the leading sport psychology journals (e.g., *Journal of Sport & Exercise Psychology*) were included in our search. These findings thus could also indicate that sport psychology is less represented than other scientific areas (physiology, medicine) in the literature. The potential underrepresentation of sport psychology should encourage sport psychologists or mental coaches to publish more of their research since there is no doubt that mental skills are an important aspect of performance in all sports.

## Strength and Limitations

The main strength of this review is the exhaustive bibliometric analysis and review across all Olympic sports. To our knowledge, no similar work is available to date. The volume of extracted articles, the clear delimitation of journals and sports and the subsequent analysis permitted us to extract information on how the “sport sciences” field is structured and organized to characterize the research body on Olympic sports and highlight sports-related differential peculiarities, developments and limitations of the scientific literature.

Some limitations must be acknowledged. First, the search was performed only in the titles of the articles and did not include searching abstracts, keywords or text. Since our aim was to compare the literature on individual sports, this method may be better suited to extracting articles related primarily to one sport without risking the inclusion of false positives that refer to specific sports only marginally or incidentally. Not all physiology or medical articles on “athletes” were included since these articles can also refer to non-specific physiological responses or mechanisms. Instead, we targeted each sport or the athletes of that sport and applied clear exclusion criteria to enhance the specificity of the search strategy. However, minor categorization inaccuracies due to the high volume of articles analyzed, particularly in the “football” and “athletics” categories, cannot be ruled out. All American and Canadian publications on football in particular were checked individually to accurately distinguish between soccer and American football. If publications could not be unambiguously classified, they were excluded. For “athletics,” the single “athlete” item in the title would have led to 10,866 publications, most of which were not related to “athletics” (Table 3). In an alternative search, specific terms related to athletics (e.g., javelin and relay) were merged, yielding a sufficiently accurate outcome. Similarly, articles with the generic term “repeated sprints” were included only if one sport was clearly mentioned in the title. There is also potential for a biased bibliometric analysis because some articles published on topics other than “exercise and sport sciences” or general medical and basic science journals could not be excluded (e.g., Olympic sports-related sociology), possibly leaving out influential works. Therefore, the present bibliometric analysis should be interpreted in light of these limitations.

Using our approach, it was not possible to differentiate research on high-level exercise from (everyday) physical activities. This limitation applies in particular to sports that occur in parallel in common everyday activities, such as walking or cycling. These categories are therefore likely overrepresented in our analysis in comparison to sports that are practiced only for competitive purposes and therefore are less frequently treated in the scientific literature. It is noteworthy that despite this bias, football still dominates the “sport sciences” field.

The absolute bibliometric is by definition correct only at the date of the search. We decided to report these absolute metrics (and not only the relative percentage values) for clarity and because it might help the reader to search beyond the top 5 articles for each sport displayed in Tables 4, 5.

One additional limitation was the descriptive nature of the analysis and the lack of statistical treatment of the data. The descriptive nature of the present article was thought to be more appropriate for the 8 main outcomes presented in the discussion. The peculiarities in significant differences in the number of citations between sport A and sport B are of negligible importance and might distract the reader from the main points.

Finally, a more fundamental criticism of the applied approach concerns the importance attached to numbers of citations generated by peer-reviewed publications as a metric for assessing the research impact (Buttner et al., 2021). For the present review, general quantitative publication metrics were used to assess only the importance of the different sports in the scientific literature in this respect. Measuring and comparing the “quality” of science between sports are challenges for future research. We are aware that the use of the top 10 most cited articles (mean, max and min citations; Figure 4) in every sport as a metric of research quality is far from optimal. Our findings show that many factors are likely involved in determining the importance of a sport-specific scientific interest, and we do not intend to understate the importance of research that is impactful in terms of policy, economics and society. Finally, it would be interesting to relate the bibliometric data presented here to the economic weight and media exposure of these sports or the number of participants in them worldwide. Such analyses may provide further insights into why certain sports are more prominently represented in the scientific literature than others. The high scientific impact of publications, for example, on football (i.e., more articles and citations), likely does not reflect “better” scientific quality than that of publications on a less prominent sports.

## Conclusions

The bibliometric analysis of all articles related to summer and winter Olympic sports published in the “sport sciences” literature provides novel insights into this research field, converging on eight key points: 1. nine sports (football, cycling, athletics, swimming, distance & marathon running, basketball, baseball, tennis, and rowing) were involved in 69% of the articles and 75% of the citations; 2. football (soccer) is the leading sport, with 19.7 and 26.3% of the total number of articles and citations, respectively; 3. team sports, especially American professional sports (i.e., baseball, basketball, and ice hockey), are the focus of prominent scientific output; 4. overall, winter sports generate comparatively minor scientific interest; 5. the greatest number of studies in the field are published in a relatively small number of “sport sciences” journals; 6. entrance into the Olympic programme may increase the scientific output of “recent” sports, although this hypothesis requires further substantiation; 7. local/cultural influences contribute to the representation of different sports in a journal's portfolio; and 8. finally, the relative distribution of six main research topics (i.e., physiology, performance, training and testing, injuries and medicine, biomechanics, and psychology) is extremely diverse across sports and provides information on the performance determinants of each sport. Overall, within the rapidly growing interdisciplinary “sport sciences” field, this bibliometric analysis provides valuable and helpful information for researchers, practitioners, and funding stakeholders to achieve future progress in the Olympic-based research agenda.

## Data Availability Statement

The original contributions presented in the study are included in the article/supplementary materials, further inquiries can be directed to the corresponding author/s.

## Author Contributions

All authors listed have made a substantial, direct and intellectual contribution to the work, and approved it for publication.

## Conflict of Interest

The authors declare that the research was conducted in the absence of any commercial or financial relationships that could be construed as a potential conflict of interest.

## Publisher's Note

All claims expressed in this article are solely those of the authors and do not necessarily represent those of their affiliated organizations, or those of the publisher, the editors and the reviewers. Any product that may be evaluated in this article, or claim that may be made by its manufacturer, is not guaranteed or endorsed by the publisher.

## References

[B1] Aagaard P. Beyer N. Simonsen E. B. Larsson B. Magnusson S. P. Kjaer M. (1998). Isokinetic muscle strength and hiking performance in elite sailors. Scand. J. Med. Sci. Sports 8, 138–144. 10.1111/j.1600-0838.1998.tb00183.x 9659673

[B2] Ackland T. R. Ong K. B. Kerr D. A. Ridge B. (2003). Morphological characteristics of Olympic sprint canoe and kayak paddlers. J. Sci. Med. Sport 6, 285–294. 10.1016/S1440-2440(03)80022-1 14609145

[B3] Agel J. Arendt E. A. Bershadsky B. (2005). Anterior cruciate ligament injury in national collegiate athletic association basketball and soccer: a 13-year review. Am. J. Sports Med. 33, 524–530. 10.1177/0363546504269937 15722283

[B4] Allen J. B. (1999). Sports medicine and sailing. Phys. Med. Rehabil. Clin. N. Am. 10, 49–65. 10.1016/S1047-9651(18)30215-8 10081052

[B5] Andersson E. Supej M. Sandbakk O. Sperlich B. Stoggl T. Holmberg H. C. (2010). Analysis of sprint cross-country skiing using a differential global navigation satellite system. Eur. J. Appl. Physiol. 110, 585–595. 10.1007/s00421-010-1535-2 20571822

[B6] Arendt E. Dick R. (1995). Knee injury patterns among men and women in collegiate basketball and soccer. NCAA data and review of literature. Am. J. Sports Med. 23, 694–701. 10.1177/036354659502300611 8600737

[B7] Artioli G. G. Franchini E. Nicastro H. Sterkowicz S. Solis M. Y. Lancha A. H. (2010). The need of a weight management control program in judo: a proposal based on the successful case of wrestling. J. Int. Soc. Sports Nutr. 7:15. 10.1186/1550-2783-7-15 20441594 PMC2876998

[B8] Aziz A. R. Chia M. Teh K. C. (2000). The relationship between maximal oxygen uptake and repeated sprint performance indices in field hockey and soccer players. J. Sports Med. Phys. Fitness. 40, 195–200. 11125761

[B9] Bahr R. Bahr I. A. (1997). Incidence of acute volleyball injuries: a prospective cohort study of injury mechanisms and risk factors. Scand. J. Med. Sci. Sports 7, 166–171. 10.1111/j.1600-0838.1997.tb00134.x 9200321

[B10] Bahr R. Lian O. Bahr I. A. (1997). A twofold reduction in the incidence of acute ankle sprains in volleyball after the introduction of an injury prevention program: a prospective cohort study. Scand. J. Med. Sci. Sports 7, 172–177. 10.1111/j.1600-0838.1997.tb00135.x 9200322

[B11] Banas M. P. Dalldorf P. G. Marquardt J. D. (1992). Skateboard and in-line skate fractures: a report of one summer's experience. J. Orthop. Trauma 6, 301–305. 10.1097/00005131-199209000-00006 1403248

[B12] Bangsbo J. Iaia F. M. Krustrup P. (2008). The Yo-Yo intermittent recovery test: a useful tool for evaluation of physical performance in intermittent sports. Sports Med. 38, 37–51. 10.2165/00007256-200838010-00004 18081366

[B13] Baranto A. Hellstrom M. Nyman R. Lundin O. Sward L. (2006). Back pain and degenerative abnormalities in the spine of young elite divers: a 5-year follow-up magnetic resonance imaging study. Knee Surg. Sports Traumatol. Arthrosc. 14, 907–914. 10.1007/s00167-005-0032-3 16416326

[B14] Barrentine S. W. Fleisig G. S. Whiteside J. A. Escamilla R. F. Andrews J. R. (1998). Biomechanics of windmill softball pitching with implications about injury mechanisms at the shoulder and elbow. J. Orthop. Sports Phys. Ther. 28, 405–415. 10.2519/jospt.1998.28.6.405 9836172

[B15] Barris S. Farrow D. Davids K. (2014). Increasing functional variability in the preparatory phase of the takeoff improves elite springboard diving performance. Res. Q. Exerc. Sport. 85, 97–106. 10.1080/02701367.2013.872220 24749241

[B16] Bassett D. R. Jr. Pucher J. Buehler R. Thompson D. L. Crouter S. E. (2008). Walking, cycling, and obesity rates in Europe, North America, and Australia. J. Phys. Act. Health 5, 795–814. 10.1123/jpah.5.6.795 19164816

[B17] Bencke J. Damsgaard R. Saekmose A. Jorgensen P. Jorgensen K. Klausen K. (2002). Anaerobic power and muscle strength characteristics of 11 years old elite and non-elite boys and girls from gymnastics, team handball, tennis and swimming. Scand. J. Med. Sci. Sports. 12, 171–178. 10.1034/j.1600-0838.2002.01128.x 12135450

[B18] Beneke R. (1995). Anaerobic threshold, individual anaerobic threshold, and maximal lactate steady state in rowing. Med. Sci. Sports Exerc. 27, 863–867. 10.1249/00005768-199506000-00010 7658947

[B19] Beneke R. Beyer T. Jachner C. Erasmus J. Hutler M. (2004). Energetics of karate kumite. Eur. J. Appl. Physiol. 92, 518–523. 10.1007/s00421-004-1073-x 15138826

[B20] Bentley D. J. Millet G. P. Vleck V. E. McNaughton L. R. (2002). Specific aspects of contemporary triathlon: implications for physiological analysis and performance. Sports Med. 32, 345–359. 10.2165/00007256-200232060-00001 11980499

[B21] Bere T. Florenes T. W. Krosshaug T. Koga H. Nordsletten L. Irving C. . (2011). Mechanisms of anterior cruciate ligament injury in World Cup alpine skiing: a systematic video analysis of 20 cases. Am. J. Sports Med. 39, 1421–1429. 10.1177/0363546511405147 21515807

[B22] Berry J. W. Romanick M. A. Koerber S. M. (2013). Injury type and incidence among elite level curlers during world championship competition. Res. Sports Med. 21, 159–163. 10.1080/15438627.2012.757229 23541102

[B23] Billat L. V. (2001). Interval training for performance: a scientific and empirical practice. Special recommendations for middle- and long-distance running. Part I: aerobic interval training. Sports Med. 31, 13–31. 10.2165/00007256-200131010-00002 11219499

[B24] Billat V. Palleja P. Charlaix T. Rizzardo P. Janel N. (1995). Energy specificity of rock climbing and aerobic capacity in competitive sport rock climbers. J. Sports Med. Phys. Fitness 35, 20–24. 7474988

[B25] Bishop D. (2008). An applied research model for the sport sciences. Sports Med. 38, 253–263. 10.2165/00007256-200838030-00005 18278985

[B26] Bishop D. Bonetti D. Dawson B. (2002). The influence of pacing strategy on VO2 and supramaximal kayak performance. Med. Sci. Sports Exerc. 34, 1041–1047. 10.1097/00005768-200206000-00022 12048335

[B27] Bladin C. Giddings P. Robinson M. (1993). Australian snowboard injury data base study. A four-year prospective study. Am. J. Sports Med. 21, 701–704. 10.1177/036354659302100511 8238711

[B28] Blajer W. Czaplicki A. (2001). Modeling and inverse simulation of somersaults on the trampoline. J. Biomech. 34, 1619–1629. 10.1016/S0021-9290(01)00139-7 11716864

[B29] Blanksby B. A. Wearne F. K. Elliott B. C. Blitvich J. D. (1997). Aetiology and occurrence of diving injuries. A review of diving safety. Sports Med. 23, 228–246. 10.2165/00007256-199723040-00003 9160480

[B30] Booth D. (2001). From bikinis to boardshorts: wahines and the paradoxes of surfing culture. J. Sport Hist. 28, 3–22. 17561560

[B31] Bradley J. L. (2009). The sports science of curling: a practical review. J. Sports Sci. Med. 8, 495–500. 24149588 PMC3761524

[B32] Bressel E. Yonker J. C. Kras J. Heath E. M. (2007). Comparison of static and dynamic balance in female collegiate soccer, basketball, and gymnastics athletes. J. Athl. Train. 42, 42–46. 17597942 PMC1896078

[B33] Bridge C. A. Ferreira da Silva Santos J. Chaabene H. Pieter W. Franchini E. (2014). Physical and physiological profiles of taekwondo athletes. Sports Med. 44, 713–733. 10.1007/s40279-014-0159-9 24549477

[B34] Brito J. Nassis G. P. Seabra A. T. Figueiredo P. (2018). Top 50 most-cited articles in medicine and science in football. BMJ Open Sport Exerc. Med. 4:e000388. 10.1136/bmjsem-2018-000388 30305923 PMC6173236

[B35] Brocherie F. Beard A. (2020). All alone we go faster, together we go further: the necessary evolution of professional and elite sporting environment to bridge the gap between research and practice. Front. Sports Act Living. 2:631147. 10.3389/fspor.2020.631147 33585813 PMC7874745

[B36] Bruce C. R. Anderson M. E. Fraser S. F. Stepto N. K. Klein R. Hopkins W. G. . (2000). Enhancement of 2000-m rowing performance after caffeine ingestion. Med. Sci. Sports Exerc. 32, 1958–1963. 10.1097/00005768-200011000-00021 11079528

[B37] Bullock N. Gulbin J. P. Martin D. T. Ross A. Holland T. Marino F. (2009). Talent identification and deliberate programming in skeleton: ice novice to Winter Olympian in 14 months. J. Sports Sci. 27, 397–404. 10.1080/02640410802549751 19191166

[B38] Bullock N. Martin D. T. Ross A. Rosemond C. D. Jordan M. J. Marino F. E. (2008). Acute effect of whole-body vibration on sprint and jumping performance in elite skeleton athletes. J. Strength Cond. Res. 22, 1371–1374. 10.1519/JSC.0b013e31816a44b5 18545165

[B39] Bullock N. Martin D. T. Ross A. Rosemond D. Marino F. E. (2007). Effect of long haul travel on maximal sprint performance and diurnal variations in elite skeleton athletes. Br. J. Sports Med. 41, 569–573. 10.1136/bjsm.2006.033233 17473002 PMC2465388

[B40] Burtscher M. Gatterer H. Flatz M. Sommersacher R. Woldrich T. Ruedl G. . (2008). Effects of modern ski equipment on the overall injury rate and the pattern of injury location in Alpine skiing. Clin. J. Sport Med. 18:355–357. 10.1097/MJT.0b013e31815fd0fe 18614888

[B41] Buttner F. Ardern C. L. Blazey P. Dastouri S. McKay H. A. Moher D. . (2021). Counting publications and citations is not just irrelevant: it is an incentive that subverts the impact of clinical research. Br. J. Sports Med. 55, 647–648. 10.1136/bjsports-2020-103146 33361277 PMC8208942

[B42] Cabello Manrique D. Gonzalez-Badillo J. J. (2003). Analysis of the characteristics of competitive badminton. Br. J. Sports Med. 37, 62–66. 10.1136/bjsm.37.1.62 12547746 PMC1724585

[B43] Caine D. Cochrane B. Caine C. Zemper E. (1989). An epidemiologic investigation of injuries affecting young competitive female gymnasts. Am. J. Sports Med. 17, 811–820. 10.1177/036354658901700616 2696378

[B44] Callow N. Hardy L. Hall C. (2001). The effects of a motivational general-mastery imagery intervention on the sport confidence of high-level badminton players. Res. Q. Exerc. Sport. 72, 389–400. 10.1080/02701367.2001.10608975 11770788

[B45] Campos F. A. Bertuzzi R. Dourado A. C. Santos V. G. Franchini E. (2012). Energy demands in taekwondo athletes during combat simulation. Eur. J. Appl. Physiol. 112, 1221–1228. 10.1007/s00421-011-2071-4 21769736

[B46] Cassell C. Benedict M. Specker B. (1996). Bone mineral density in elite 7- to 9-yr-old female gymnasts and swimmers. Med. Sci. Sports Exerc. 28, 1243–1246. 10.1097/00005768-199610000-00006 8897380

[B47] Causer J. Bennett S. J. Holmes P. S. Janelle C. M. Williams A. M. (2010). Quiet eye duration and gun motion in elite shotgun shooting. Med. Sci. Sports Exerc. 42, 1599–1608. 10.1249/MSS.0b013e3181d1b059 20139787

[B48] Chaabene H. Hachana Y. Franchini E. Mkaouer B. Chamari K. (2012). Physical and physiological profile of elite karate athletes. Sports Med. 42, 829–843. 10.1007/BF03262297 22901041

[B49] Chelly S. M. Denis C. (2001). Leg power and hopping stiffness: relationship with sprint running performance. Med. Sci. Sports Exerc. 33, 326–333. 10.1097/00005768-200102000-00024 11224825

[B50] Cochrane D. J. Stannard S. R. (2005). Acute whole body vibration training increases vertical jump and flexibility performance in elite female field hockey players. Br. J. Sports Med. 39, 860–865. 10.1136/bjsm.2005.019950 16244199 PMC1725065

[B51] Conley D. L. Krahenbuhl G. S. (1980). Running economy and distance running performance of highly trained athletes. Med. Sci. Sports Exerc. 12, 357–360. 10.1249/00005768-198012050-00010 7453514

[B52] Costill D. L. Flynn M. G. Kirwan J. P. Houmard J. A. Mitchell J. B. Thomas R. . (1988). Effects of repeated days of intensified training on muscle glycogen and swimming performance. Med. Sci. Sports Exerc. 20, 249–254. 10.1249/00005768-198806000-00006 3386503

[B53] Costill D. L. Kovaleski J. Porter D. Kirwan J. Fielding R. King D. (1985). Energy expenditure during front crawl swimming: predicting success in middle-distance events. Int. J. Sports Med. 6, 266–270. 10.1055/s-2008-1025849 4055188

[B54] Coutinho L. A. Porto C. P. Pierucci A. P. (2016). Critical evaluation of food intake and energy balance in young modern pentathlon athletes: a cross-sectional study. J. Int. Soc. Sports Nutr. 13:15. 10.1186/s12970-016-0127-x 27042167 PMC4818861

[B55] Coutts A. J. (2016). Working fast and working slow: the benefits of embedding research in high performance sport. Int. J. Sports Physiol. Perform. 11, 1–2. 10.1123/IJSPP.2015-0781 26752203

[B56] Coutts A. J. Wallace L. K. Slattery K. M. (2007). Monitoring changes in performance, physiology, biochemistry, and psychology during overreaching and recovery in triathletes. Int. J. Sports Med. 28, 125–134. 10.1055/s-2006-924146 16835823

[B57] Coyle E. F. Feltner M. E. Kautz S. A. Hamilton M. T. Montain S. J. Baylor A. M. . (1991). Physiological and biomechanical factors associated with elite endurance cycling performance. Med. Sci. Sports Exerc. 23, 93–107. 10.1249/00005768-199101000-00015 1997818

[B58] Coyle E. F. Sidossis L. S. Horowitz J. F. Beltz J. D. (1992). Cycling efficiency is related to the percentage of type I muscle fibers. Med. Sci. Sports Exerc. 24, 782–788. 10.1249/00005768-199207000-00008 1501563

[B59] Craig A. B. Jr. Pendergast D. R. (1979). Relationships of stroke rate, distance per stroke, and velocity in competitive swimming. Med. Sci. Sports. 11, 278–283. 10.1249/00005768-197901130-00011 522640

[B60] Crockett H. C. Gross L. B. Wilk K. E. Schwartz M. L. Reed J. O'Mara J. . (2002). Osseous adaptation and range of motion at the glenohumeral joint in professional baseball pitchers. Am. J. Sports Med. 30, 20–26. 10.1177/03635465020300011701 11798991

[B61] Crossland B. W. Hartman J. E. Kilgore J. L. Hartman M. J. Kaus J. M. (2011). Upper-body anthropometric and strength measures and their relationship to start time in elite luge athletes. J. Strength Cond. Res. 25, 2639–2644. 10.1519/JSC.0b013e318207ed7a 21873904

[B62] Cummings R. S. Jr. Shurland A. T. Prodoehl J. A. Moody K. Sherk H. H. (1997). Injuries in the sport of luge. Epidemiol. Analys. Am J Sports Med. 25, 508–513. 10.1177/036354659702500414 9240985

[B63] Da Roza T. Brandao S. Mascarenhas T. Jorge R. N. Duarte J. A. (2015). Volume of training and the ranking level are associated with the leakage of urine in young female trampolinists. Clin. J. Sport Med. 25, 270–275. 10.1097/JSM.0000000000000129 25010151

[B64] Dabnichki P. Avital E. (2006). Influence of the postion of crew members on aerodynamics performance of two-man bobsleigh. J. Biomech. 39, 2733–2742. 10.1016/j.jbiomech.2005.10.011 16298374

[B65] Dadswell C. E. Payton C. Holmes P. Burden A. (2013). Biomechanical analysis of the change in pistol shooting format in modern pentathlon. J. Sports Sci. 31, 1294–1301. 10.1080/02640414.2013.777762 23496339

[B66] Degoutte F. Jouanel P. Begue R. J. Colombier M. Lac G. Pequignot J. M. . (2006). Food restriction, performance, biochemical, psychological, and endocrine changes in judo athletes. Int. J. Sports Med. 27, 9–18. 10.1055/s-2005-837505 16388436

[B67] Degoutte F. Jouanel P. Filaire E. (2003). Energy demands during a judo match and recovery. Br. J. Sports Med. 37, 245–249. 10.1136/bjsm.37.3.245 12782550 PMC1724647

[B68] Devienne M. F. Guezennec C. Y. (2000). Energy expenditure of horse riding. Eur. J. Appl. Physiol. 82, 499–503. 10.1007/s004210000207 10985607

[B69] Di Russo F. Pitzalis S. Aprile T. Spinelli D. (2005). Effect of practice on brain activity: an investigation in top-level rifle shooters. Med. Sci. Sports Exerc. 37, 1586–1593. 10.1249/01.mss.0000177458.71676.0d 16177612

[B70] Doria C. Veicsteinas A. Limonta E. Maggioni M. A. Aschieri P. Eusebi F. . (2009). Energetics of karate (kata and kumite techniques) in top-level athletes. Eur. J. Appl. Physiol. 107, 603–610. 10.1007/s00421-009-1154-y 19711097

[B71] Dupont G. Millet G. P. Guinhouya C. Berthoin S. (2005). Relationship between oxygen uptake kinetics and performance in repeated running sprints. Eur. J. Appl. Physiol. Occup. Physiol. 95:27–34. 10.1007/s00421-005-1382-8 15976999

[B72] Elferink-Gemser M. T. Visscher C. Lemmink K. A. Mulder T. W. (2004). Relation between multidimensional performance characteristics and level of performance in talented youth field hockey players. J. Sports Sci. 22, 1053–1063. 10.1080/02640410410001729991 15801499

[B73] Eliasson K. Larsson T. Mattsson E. (2002). Prevalence of stress incontinence in nulliparous elite trampolinists. Scand. J. Med. Sci. Sports. 12, 106–110. 10.1034/j.1600-0838.2002.120207.x 12121428

[B74] Ellenbecker T. S. Roetert E. P. Bailie D. S. Davies G. J. Brown S. W. (2002). Glenohumeral joint total rotation range of motion in elite tennis players and baseball pitchers. Med. Sci. Sports Exerc. 34, 2052–2056. 10.1097/00005768-200212000-00028 12471315

[B75] Era P. Konttinen N. Mehto P. Saarela P. Lyytinen H. (1996). Postural stability and skilled performance–a study on top-level and naive rifle shooters. J. Biomech. 29, 301–306. 10.1016/0021-9290(95)00066-6 8850636

[B76] Ertan H. Kentel B. Tumer S. T. Korkusuz F. (2003). Activation patterns in forearm muscles during archery shooting. Hum. Mov. Sci. 22, 37–45. 10.1016/S0167-9457(02)00176-8 12623179

[B77] Ettlinger C. F. Johnson R. J. Shealy J. E. (1995). A method to help reduce the risk of serious knee sprains incurred in alpine skiing. Am. J. Sports Med. 23, 531–537. 10.1177/036354659502300503 8526266

[B78] Falco C. Alvarez O. Castillo I. Estevan I. Martos J. Mugarra F. . (2009). Influence of the distance in a roundhouse kick's execution time and impact force in Taekwondo. J. Biomech. 42, 242–248. 10.1016/j.jbiomech.2008.10.041 19124126

[B79] Farley O. R. Harris N. K. Kilding A. E. (2012). Physiological demands of competitive surfing. J. Strength Cond. Res. 26, 1887–1896. 10.1519/JSC.0b013e3182392c4b 21986691

[B80] Faude O. Meyer T. Rosenberger F. Fries M. Huber G. Kindermann W. (2007). Physiological characteristics of badminton match play. Eur. J. Appl. Physiol. 100, 479–485. 10.1007/s00421-007-0441-8 17473928

[B81] Fernandez J. Mendez-Villanueva A. Pluim B. M. (2006). Intensity of tennis match play. Br. J. Sports Med. 40, 387–391; discussion 91. 10.1136/bjsm.2005.023168 16632566 PMC2653872

[B82] Ferretti A. Papandrea P. Conteduca F. Mariani P. P. (1992). Knee ligament injuries in volleyball players. Am. J. Sports Med. 20, 203–207. 10.1177/036354659202000219 1558250

[B83] Fleisig G. S. Andrews J. R. Dillman C. J. Escamilla R. F. (1995). Kinetics of baseball pitching with implications about injury mechanisms. Am. J. Sports Med. 23, 233–239. 10.1177/036354659502300218 7778711

[B84] Fleisig G. S. Barrentine S. W. Zheng N. Escamilla R. F. Andrews J. R. (1999). Kinematic and kinetic comparison of baseball pitching among various levels of development. J. Biomech. 32, 1371–1375. 10.1016/S0021-9290(99)00127-X 10569718

[B85] Flik K. Lyman S. Marx R. G. (2005). American collegiate men's ice hockey: an analysis of injuries. Am. J. Sports Med. 33, 183–187. 10.1177/0363546504267349 15701603

[B86] Florenes T. W. Bere T. Nordsletten L. Heir S. Bahr R. (2009). Injuries among male and female World Cup alpine skiers. Br. J. Sports Med. 43, 973–978. 10.1136/bjsm.2009.068759 19945979

[B87] Ford K. R. Myer G. D. Hewett T. E. (2003). Valgus knee motion during landing in high school female and male basketball players. Med. Sci. Sports Exerc. 35, 1745–1750. 10.1249/01.MSS.0000089346.85744.D9 14523314

[B88] Forsman L. Eriksson A. (2001). Skateboarding injuries of today. Br. J. Sports Med. 35, 325–328. 10.1136/bjsm.35.5.325 11579065 PMC1724407

[B89] Foster C. Rundell K. W. Snyder A. C. Stray-Gundersen J. Kemkers G. Thometz N. . (1999). Evidence for restricted muscle blood flow during speed skating. Med. Sci. Sports Exerc. 31, 1433–1440. 10.1097/00005768-199910000-00012 10527316

[B90] Fountain J. L. Meyers M. C. (1996). Skateboarding injuries. Sports Med. 22, 360–366. 10.2165/00007256-199622060-00004 8969014

[B91] Franchini E. Del Vecchio F. B. Matsushigue K. A. Artioli G. G. (2011). Physiological profiles of elite judo athletes. Sports Med. 41, 147–166. 10.2165/11538580-000000000-00000 21244106

[B92] Furness J. Hing W. Walsh J. Abbott A. Sheppard J. M. Climstein M. (2015). Acute injuries in recreational and competitive surfers: incidence, severity, location, type, and mechanism. Am. J. Sports Med. 43:, 1246–1254. 10.1177/0363546514567062 25646362

[B93] Garcia-Pallares J. Sanchez-Medina L. Carrasco L. Diaz A. Izquierdo M. (2009). Endurance and neuromuscular changes in world-class level kayakers during a periodized training cycle. Eur. J. Appl. Physiol. 106, 629–638. 10.1007/s00421-009-1061-2 19396614

[B94] Garhammer J. (1980). Power production by Olympic weightlifters. Med. Sci. Sports Exerc. 12, 54–60. 10.1249/00005768-198021000-00011 7392903

[B95] Gau L. S. (2013). Trends and topics in sports research in the Social Science Citation Index from 1993 to 2008. Percept. Mot. Skills. 116, 305–314. 10.2466/30.03.PMS.116.1.305-314 23829156

[B96] Giombini A. Dragoni S. Di Cesare A. Di Cesare M. Del Buono A. Maffulli N. (2013). Asymptomatic Achilles, patellar, and quadriceps tendinopathy: a longitudinal clinical and ultrasonographic study in elite fencers. Scand. J. Med. Sci. Sports 23, 311–316. 10.1111/j.1600-0838.2011.01400.x 22092963

[B97] Gleeson M. McDonald W. A. Pyne D. B. Cripps A. W. Francis J. L. Fricker P. A. . (1999). Salivary IgA levels and infection risk in elite swimmers. Med. Sci. Sports Exerc. 31, 67–73. 10.1097/00005768-199901000-00012 9927012

[B98] Gorostiaga E. M. Granados C. Ibanez J. Izquierdo M. (2005). Differences in physical fitness and throwing velocity among elite and amateur male handball players. Int. J. Sports Med. 26, 225–232. 10.1055/s-2004-820974 15776339

[B99] Gould D. Eklund R. C. Jackson S. A. (1993b). Coping strategies used by U.S. Olympic wrestlers. Res. Q. Exerc. Sport 64, 83–93. 10.1080/02701367.1993.10608782 8451537

[B100] Gould D. Finch L. M. Jackson S. A. (1993a). Coping strategies used by national champion figure skaters. Res. Q. Exerc. Sport. 64, 453–468. 10.1080/02701367.1993.10607599 8278672

[B101] Grant S. Hynes V. Whittaker A. Aitchison T. (1996). Anthropometric, strength, endurance and flexibility characteristics of elite and recreational climbers. J. Sports Sci. 14, 301–309. 10.1080/02640419608727715 8887209

[B102] Gregory J. Johns D. P. Walls J. T. (2007). Relative vs. absolute physiological measures as predictors of mountain bike cross-country race performance. J. Strength Cond. Res. 21, 17–22. 10.1519/00124278-200702000-00004 17313256

[B103] Grimsmo J. Grundvold I. Maehlum S. Arnesen H. (2010). High prevalence of atrial fibrillation in long-term endurance cross-country skiers: echocardiographic findings and possible predictors–a 28-30 years follow-up study. Eur. J. Cardiovasc. Prev. Rehabil. 17, 100–105. 10.1097/HJR.0b013e32833226be 20065854

[B104] Haff G. G. Carlock J. M. Hartman M. J. Kilgore J. L. Kawamori N. Jackson J. R. . (2005). Force-time curve characteristics of dynamic and isometric muscle actions of elite women olympic weightlifters. J. Strength Cond. Res. 19, 741–748. 10.1519/00124278-200511000-00004 16287343

[B105] Hagerman F. C. (1984). Applied physiology of rowing. Sports Med. 1, 303–326. 10.2165/00007256-198401040-00005 6390606

[B106] Hall C. J. Lane A. M. (2001). Effects of rapid weight loss on mood and performance among amateur boxers. Br. J. Sports Med. 35, 390–395. 10.1136/bjsm.35.6.390 11726472 PMC1724425

[B107] Haralambie G. Cerny F. J. Huber G. (1976). Serum enzyme levels after bobsled racing. J. Sports Med. Phys. Fitness. 16, 54–56. 1263475

[B108] Haugen T. Paulsen G. Seiler S. Sandbakk O. (2018). New records in human power. Int. J. Sports Physiol. Perform. 13, 678–686. 10.1123/ijspp.2017-0441 28872385

[B109] Hebert-Losier K. Zinner C. Platt S. Stoggl T. Holmberg H. C. (2017). Factors that influence the performance of elite sprint cross-country skiers. Sports Med. 47, 319–342. 10.1007/s40279-016-0573-2 27334280 PMC5266777

[B110] Heinicke K. Heinicke I. Schmidt W. Wolfarth B. (2005). A three-week traditional altitude training increases hemoglobin mass and red cell volume in elite biathlon athletes. Int. J. Sports Med. 26, 350–355. 10.1055/s-2004-821052 15895317

[B111] Heller M. O. Bergmann G. Deuretzbacher G. Durselen L. Pohl M. Claes L. . (2001). Musculo-skeletal loading conditions at the hip during walking and stair climbing. J. Biomech. 34, 883–893. 10.1016/S0021-9290(01)00039-2 11410172

[B112] Hermansen L. Saltin B. (1969). Oxygen uptake during maximal treadmill and bicycle exercise. J Appl Physiol. 26, 31–37. 10.1152/jappl.1969.26.1.31 5762873

[B113] Herzog W. Guimaraes A. C. Anton M. G. Carter-Erdman K. A. (1991). Moment-length relations of rectus femoris muscles of speed skaters/cyclists and runners. Med. Sci. Sports Exerc. 23, 1289–1296. 10.1249/00005768-199111000-00015 1766346

[B114] Higham D. G. Pyne D. B. Anson J. M. Eddy A. (2012). Movement patterns in rugby sevens: effects of tournament level, fatigue and substitute players. J. Sci. Med. Sport 15, 277–282. 10.1016/j.jsams.2011.11.256 22188846

[B115] Higham D. G. Pyne D. B. Anson J. M. Eddy A. (2013). Physiological, anthropometric, and performance characteristics of rugby sevens players. Int. J. Sports Physiol. Perform. 8, 19–27. 10.1123/ijspp.8.1.19 22868376

[B116] Hill A. V. (1925). The physiological basis of athletic records. Nature 116, 544–548. 10.1038/116544a0

[B117] Hodson R. (2021). Sports science. Nature 592:S1. 10.1038/d41586-021-00814-5 33790453

[B118] Hoff J. Helgerud J. Wisloff U. (1999). Maximal strength training improves work economy in trained female cross-country skiers. Med. Sci. Sports Exerc. 31, 870–877. 10.1097/00005768-199906000-00016 10378915

[B119] Hoffman M. D. Gilson P. M. Westenburg T. M. Spencer W. A. (1992). Biathlon shooting performance after exercise of different intensities. Int. J. Sports Med. 13, 270–273. 10.1055/s-2007-1021265 1601564

[B120] Holmberg H. C. Lindinger S. Stoggl T. Eitzlmair E. Muller E. (2005). Biomechanical analysis of double poling in elite cross-country skiers. Med. Sci. Sports Exerc. 37, 807–818. 10.1249/01.MSS.0000162615.47763.C8 15870635

[B121] Hori N. Newton R. U. Andrews W. A. Kawamori N. McGuigan M. R. Nosaka K. (2008). Does performance of hang power clean differentiate performance of jumping, sprinting, and changing of direction? J. Strength Cond. Res. 22, 412–418. 10.1519/JSC.0b013e318166052b 18550955

[B122] Hosseini A. H. Lifshitz J. (2009). Brain injury forces of moderate magnitude elicit the fencing response. Med. Sci. Sports Exerc. 41, 1687–1697. 10.1249/MSS.0b013e31819fcd1b 19657303 PMC11421656

[B123] Hristovski R. Davids K. Araujo D. Button C. (2006). How boxers decide to punch a target: emergent behaviour in nonlinear dynamical movement systems. J. Sports Sci. Med. 5, 60–73. 24357978 PMC3863932

[B124] Hume P. A. Chalmers D. J. Wilson B. D. (1996). Trampoline injury in New Zealand: emergency care. Br. J. Sports Med. 30, 327–330. 10.1136/bjsm.30.4.327 9015596 PMC1332419

[B125] Hume P. A. Keogh J. Reid D. (2005). The role of biomechanics in maximising distance and accuracy of golf shots. Sports Med. 35, 429–449. 10.2165/00007256-200535050-00005 15896091

[B126] Hunter J. P. Marshall R. N. McNair P. J. (2005). Relationships between ground reaction force impulse and kinematics of sprint-running acceleration. J. Appl. Biomech. 21, 31–43. 10.1123/jab.21.1.31 16131703

[B127] Iino Y. Kojima T. (2009). Kinematics of table tennis topspin forehands: effects of performance level and ball spin. J. Sports Sci. 27, 1311–1321. 10.1080/02640410903264458 19746298

[B128] Impellizzeri F. M. Rampinini E. Coutts A. J. Sassi A. Marcora S. M. (2004). Use of RPE-based training load in soccer. Med. Sci. Sports Exerc. 36, 1042–1047. 10.1249/01.MSS.0000128199.23901.2F 15179175

[B129] Jeukendrup A. E. (2011). Nutrition for endurance sports: marathon, triathlon, and road cycling. J. Sports Sci. 29(Suppl. 1):S91–S99. 10.1080/02640414.2011.610348 21916794

[B130] Jones A. M. Kirby B. S. Clark I. E. Rice H. M. Fulkerson E. Wylie L. J. . (2021). Physiological demands of running at 2-hour marathon race pace. J Appl Physiol. 130, 369–379. 10.1152/japplphysiol.00647.2020 33151776

[B131] Kazemi M. Waalen J. Morgan C. White A. R. (2006). A profile of olympic taekwondo competitors. J. Sports Sci. Med. 5, 114–121. 24357983 PMC3863920

[B132] Kelly P. Kahlmeier S. Gotschi T. Orsini N. Richards J. Roberts N. . (2014). Systematic review and meta-analysis of reduction in all-cause mortality from walking and cycling and shape of dose response relationship. Int. J. Behav. Nutr. Phys. Act. 11:132. 10.1186/s12966-014-0132-x 25344355 PMC4262114

[B133] Khatra O. Shadgan A. Taunton J. Pakravan A. Shadgan B. (2021). A bibliometric analysis of the top cited articles in sports and exercise medicine. Orthopaedic J. Sports Med. 9:2325967120969902. 10.1177/2325967120969902 33553441 PMC7841868

[B134] Kim S. Endres N. K. Johnson R. J. Ettlinger C. F. Shealy J. E. (2012). Snowboarding injuries: trends over time and comparisons with alpine skiing injuries. Am. J. Sports Med. 40, 770–776. 10.1177/0363546511433279 22268231

[B135] Knudson D. V. (2011). Authorship and sampling practice in selected biomechanics and sports science journals. Percept. Mot. Skills. 112, 838–844. 10.2466/17.PMS.112.3.838-844 21853773

[B136] Koga H. Nakamae A. Shima Y. Iwasa J. Myklebust G. Engebretsen L. . (2010). Mechanisms for noncontact anterior cruciate ligament injuries: knee joint kinematics in 10 injury situations from female team handball and basketball. Am. J. Sports Med. 38, 2218–2225. 10.1177/0363546510373570 20595545

[B137] Kolt G. S. Kirkby R. J. (1999). Epidemiology of injury in elite and subelite female gymnasts: a comparison of retrospective and prospective findings. Br. J. Sports Med. 33, 312–318. 10.1136/bjsm.33.5.312 10522632 PMC1756196

[B138] Kondric M. Zagatto A. M. Sekulic D. (2013). The physiological demands of table tennis: a review. J. Sports Sci. Med. 12, 362–370. 24149139 PMC3772576

[B139] Kraemer W. J. Fry A. C. Rubin M. R. Triplett-McBride T. Gordon S. E. Koziris L. P. . (2001). Physiological and performance responses to tournament wrestling. Med. Sci. Sports Exerc. 33, 1367–1378. 10.1097/00005768-200108000-00019 11474340

[B140] Krahenbuhl G. S. Wells C. L. Brown C. H. Ward P. E. (1979). Characteristics of national and world class female pentathletes. Med. Sci. Sports 11, 20–23. 481151

[B141] Kroncke E. L. Niedfeldt M. W. Young C. C. (2008). Use of protective equipment by adolescents in inline skating, skateboarding, and snowboarding. Clin. J. Sport Med. 18, 38–43. 10.1097/JSM.0b013e318160c044 18185037

[B142] Krosshaug T. Nakamae A. Boden B. P. Engebretsen L. Smith G. Slauterbeck J. R. . (2007). Mechanisms of anterior cruciate ligament injury in basketball: video analysis of 39 cases. Am. J. Sports Med. 35, 359–367. 10.1177/0363546506293899 17092928

[B143] Kuitunen S. Komi P. V. Kyrolainen H. (2002). Knee and ankle joint stiffness in sprint running. Med. Sci. Sports Exerc. 34, 166–173. 10.1097/00005768-200201000-00025 11782663

[B144] Kuntze G. Mansfield N. Sellers W. (2010). A biomechanical analysis of common lunge tasks in badminton. J. Sports Sci. 28, 183–191. 10.1080/02640410903428533 20391092

[B145] Kusma M. Jung J. Dienst M. Goedde S. Kohn D. Seil R. (2004). Arthroscopic treatment of an avulsion fracture of the ligamentum teres of the hip in an 18-year-old horse rider. Arthroscopy. 20(Suppl. 2), 64–66. 10.1016/j.arthro.2004.04.041 15243428

[B146] Landers D. M. Petruzzello S. J. Salazar W. Crews D. J. Kubitz K. A. Gannon T. L. . (1991). The influence of electrocortical biofeedback on performance in pre-elite archers. Med. Sci. Sports Exerc. 23, 123–129. 10.1249/00005768-199101000-00018 1997806

[B147] Le Meur Y. Dorel S. Baup Y. Guyomarch J. P. Roudaut C. Hausswirth C. (2012). Physiological demand and pacing strategy during the new combined event in elite pentathletes. Eur. J. Appl. Physiol. 112, 2583–2593. 10.1007/s00421-011-2235-2 22081048

[B148] Le Meur Y. Hausswirth C. Abbiss C. Baup Y. Dorel S. (2010). Performance factors in the new combined event of modern pentathlon. J. Sports Sci. 28, 1111–1116. 10.1080/02640414.2010.497816 20686991

[B149] Lee H. Martin D. T. Anson J. M. Grundy D. Hahn A. G. (2002). Physiological characteristics of successful mountain bikers and professional road cyclists. J. Sports Sci. 20, 1001–1008. 10.1080/026404102321011760 12477010

[B150] Lee I. M. Buchner D. M. (2008). The importance of walking to public health. Med. Sci. Sports Exerc. 40(7 Suppl.), S512–S518. 10.1249/MSS.0b013e31817c65d0 18562968

[B151] Lembert S. Schachner O. Raschner C. (2011). Development of a measurement and feedback training tool for the arm strokes of high-performance luge athletes. J. Sports Sci. 29, 1593–1601. 10.1080/02640414.2011.608433 22077383

[B152] Leroyer P. Van Hoecke J. Helal J. N. (1993). Biomechanical study of the final push-pull in archery. J. Sports Sci. 11, 63–69. 10.1080/02640419308729965 8450588

[B153] Lewis R. M. Redzic M. Thomas D. T. (2013). The effects of season-long vitamin D supplementation on collegiate swimmers and divers. Int. J. Sport Nutr. Exerc. Metab. 23, 431–440. 10.1123/ijsnem.23.5.431 23475128 PMC4395005

[B154] Liow D. K. Hopkins W. G. (2003). Velocity specificity of weight training for kayak sprint performance. Med. Sci. Sports Exerc. 35, 1232–1237. 10.1249/01.MSS.0000074450.97188.CF 12840647

[B155] Lippi G. Guidi G. C. Nevill A. Boreham C. (2008). The growing trend of scientific interest in sports science research. J. Sports Sci. 26, 1–2. 10.1080/02640410701705108 17943593

[B156] Lloyd R. G. (1987). Riding and other equestrian injuries: considerable severity. Br. J. Sports Med. 21, 22–24. 10.1136/bjsm.21.1.22 3580722 PMC1478604

[B157] Lopes A. D. Alouche S. R. (2016). Two-man bobsled push start analysis. J. Hum. Kinet. 50, 63–70. 10.1515/hukin-2015-0143 28149342 PMC5260641

[B158] Loze G. M. Collins D. Holmes P. S. (2001). Pre-shot EEG alpha-power reactivity during expert air-pistol shooting: a comparison of best and worst shots. J. Sports Sci. 19, 727–733. 10.1080/02640410152475856 11522148

[B159] Lupo C. Tessitore A. Minganti C. Capranica L. (2010). Notational analysis of elite and sub-elite water polo matches. J. Strength Cond. Res. 24, 223–229. 10.1519/JSC.0b013e3181c27d36 19996771

[B160] Lyman S. Fleisig G. S. Andrews J. R. Osinski E. D. (2002). Effect of pitch type, pitch count, and pitching mechanics on risk of elbow and shoulder pain in youth baseball pitchers. Am. J. Sports Med. 30, 463–468. 10.1177/03635465020300040201 12130397

[B161] Mackinnon L. T. Ginn E. Seymour G. J. (1993). Decreased salivary immunoglobulin A secretion rate after intense interval exercise in elite kayakers. Eur. J. Appl. Physiol. Occup. Physiol. 67, 180–184. 10.1007/BF00376664 8223526

[B162] MacLeod H. Morris J. Nevill A. Sunderland C. (2009). The validity of a non-differential global positioning system for assessing player movement patterns in field hockey. J. Sports Sci. 27, 121–128. 10.1080/02640410802422181 19058089

[B163] MacRae H.-H. Hise K. J. Allen P. J. (2000). Effects of front and dual suspension mountain bike systems on uphill cycling performance. Med. Sci. Sports Exerc. 32, 1276–1280. 10.1097/00005768-200007000-00014 10912893

[B164] Mann D. L. Littke N. (1989). Shoulder injuries in archery. Can. J. Sport Sci. 14, 85–92. 2736447

[B165] Marshall S. W. Hamstra-Wright K. L. Dick R. Grove K. A. Agel J. (2007). Descriptive epidemiology of collegiate women's softball injuries: National Collegiate Athletic Association Injury Surveillance System, 1988-1989 through 2003-2004. J. Athl. Train. 42, 286–294. 17710178 PMC1941294

[B166] Martinent G. Ferrand C. (2009). A naturalistic study of the directional interpretation process of discrete emotions during high-stakes table tennis matches. J. Sport Exerc. Psychol. 31, 318–336. 10.1123/jsep.31.3.318 19798996

[B167] Matsushigue K. A. Hartmann K. Franchini E. (2009). Taekwondo: Physiological responses and match analysis. J. Strength Cond. Res. 23, 1112–1117. 10.1519/JSC.0b013e3181a3c597 19528839

[B168] McCrory P. Turner M. (2005). Equestrian injuries. Med. Sport Sci. 48, 8–17. 10.1159/000084280 16247251

[B169] McMaster W. C. Long S. C. Caiozzo V. J. (1991). Isokinetic torque imbalances in the rotator cuff of the elite water polo player. Am. J. Sports Med. 19, 72–75. 10.1177/036354659101900112 2008934

[B170] Mendez-Villanueva A. Bishop D. Hamer P. (2006). Activity profile of world-class professional surfers during competition: a case study. J. Strength Cond. Res. 20, 477–482. 10.1519/00124278-200608000-00004 16937958

[B171] Mermier C. M. Janot J. M. Parker D. L. Swan J. G. (2000). Physiological and anthropometric determinants of sport climbing performance. Br. J. Sports Med. 34, 359–365. 10.1136/bjsm.34.5.359 11049146 PMC1756253

[B172] Mero A. Komi P. V. Gregor R. J. (1992). Biomechanics of sprint running. A review. Sports Med. 13, 376–392. 10.2165/00007256-199213060-00002 1615256

[B173] Millet G. P. Giulianotti R. (2019). Sports and active living are medicine, and education, happiness, performance, business, innovation, and culture for a sustainable world. Front. Sports Act Living 1:1. 10.3389/fspor.2019.00001 33344925 PMC7739756

[B174] Millet G. P. Jornet K. (2019). On top to the top-acclimatization strategy for the “fastest known time” to mount everest. Int. J. Sports Physiol. Perform. 10, 1–4. 10.1123/ijspp.2018-0931 30958056

[B175] Millet G. P. Vleck V. E. Bentley D. J. (2009). Physiological differences between cycling and running: lessons from triathletes. Sports Med. 39, 179–206. 10.2165/00007256-200939030-00002 19290675

[B176] Millet G. Y. Lepers R. (2004). Alterations of neuromuscular function after prolonged running, cycling and skiing exercises. Sports Med. 34, 105–116. 10.2165/00007256-200434020-00004 14965189

[B177] Mishra A. K. Skrepnik N. V. Edwards S. G. Jones G. L. Sampson S. Vermillion D. A. . (2014). Efficacy of platelet-rich plasma for chronic tennis elbow: a double-blind, prospective, multicenter, randomized controlled trial of 230 patients. Am. J. Sports Med. 42, 463–471. 10.1177/0363546513494359 23825183

[B178] Mohr M. Krustrup P. Bangsbo J. (2003). Match performance of high-standard soccer players with special reference to development of fatigue. J. Sports Sci. 21, 519–528. 10.1080/0264041031000071182 12848386

[B179] Mononen K. Konttinen N. Viitasalo J. Era P. (2007). Relationships between postural balance, rifle stability and shooting accuracy among novice rifle shooters. Scand. J. Med. Sci. Sports 17, 180–185. 17394480 10.1111/j.1600-0838.2006.00549.x

[B180] Morgan W. P. Costill D. L. Flynn M. G. Raglin J. S. O'Connor P. J. (1988). Mood disturbance following increased training in swimmers. Med. Sci. Sports Exerc. 20, 408–414. 10.1249/00005768-198808000-00014 3173050

[B181] Mori S. Ohtani Y. Imanaka K. (2002). Reaction times and anticipatory skills of karate athletes. Hum. Mov. Sci. 21, 213–230. 10.1016/S0167-9457(02)00103-3 12167300

[B182] Mossner M. Hasler M. Schindelwig K. Kaps P. Nachbauer W. (2011). An approximate simulation model for initial luge track design. J. Biomech. 44, 892–896. 10.1016/j.jbiomech.2010.12.001 21185562

[B183] Mountjoy M. Alonso J. M. Bergeron M. F. Dvorak J. Miller S. Migliorini S. . (2012). Hyperthermic-related challenges in aquatics, athletics, football, tennis and triathlon. Br. J. Sports Med. 46, 800–804. 10.1136/bjsports-2012-091272 22906783

[B184] Mountjoy M. Junge A. Alonso J. M. Clarsen B. Pluim B. M. Shrier I. . (2016). Consensus statement on the methodology of injury and illness surveillance in FINA (aquatic sports). Br. J. Sports Med. 50, 590–596. 10.1136/bjsports-2015-095686 26614761

[B185] Mujika I. Sharma A. P. Stellingwerff T. (2019). Contemporary periodization of altitude training for elite endurance athletes: a narrative review. Sports Med. 49, 1651–1669. 10.1007/s40279-019-01165-y 31452130

[B186] Myklebust G. Engebretsen L. Braekken I. H. Skjolberg A. Olsen O. E. Bahr R. (2003). Prevention of anterior cruciate ligament injuries in female team handball players: a prospective intervention study over three seasons. Clin. J. Sport Med. 13, 71–78. 10.1097/00042752-200303000-00002 12629423

[B187] Myklebust G. Maehlum S. Holm I. Bahr R. (1998). A prospective cohort study of anterior cruciate ligament injuries in elite Norwegian team handball. Scand. J. Med. Sci. Sports 8, 149–153. 10.1111/j.1600-0838.1998.tb00185.x 9659675

[B188] Nathanson A. Bird S. Dao L. Tam-Sing K. (2007). Competitive surfing injuries: a prospective study of surfing-related injuries among contest surfers. Am. J. Sports Med. 35, 113–117. 10.1177/0363546506293702 17021312

[B189] Nathanson A. T. Reinert S. E. (1999). Windsurfing injuries: results of a paper- and Internet-based survey. Wilderness Environ. Med. 10, 218–225. 10.1580/1080-6032(1999)010[0218:WIROAP]2.3.CO;2 10628281

[B190] Newton R. U. Kraemer W. J. Hakkinen K. (1999). Effects of ballistic training on preseason preparation of elite volleyball players. Med. Sci. Sports Exerc. 31, 323–330. 10.1097/00005768-199902000-00017 10063823

[B191] Nieman D. C. Johanssen L. M. Lee J. W. Arabatzis K. (1990). Infectious episodes in runners before and after the Los Angeles Marathon. J. Sports Med. Phys. Fitness. 30, 316–328. 2266764

[B192] Nimphius S. McGuigan M. R. Newton R. U. (2010). Relationship between strength, power, speed, and change of direction performance of female softball players. J. Strength Cond. Res. 24, 885–895. 10.1519/JSC.0b013e3181d4d41d 20300038

[B193] Nirschl R. P. (1992). Elbow tendinosis/tennis elbow. Clin. Sports Med. 11, 851–870. 10.1016/S0278-5919(20)30489-0 1423702

[B194] Nysted M. Drogset J. O. (2006). Trampoline injuries. Br. J. Sports Med. 40, 984–987. 10.1136/bjsm.2006.029009 17000711 PMC2577468

[B195] O' Donoghue P. Ingram B. (2001). A notational analysis of elite tennis strategy. J. Sports Sci. 19, 107–115. 10.1080/026404101300036299 11217009

[B196] Oja P. Titze S. Bauman A. de Geus B. Krenn P. Reger-Nash B. . (2011). Health benefits of cycling: a systematic review. Scand. J. Med. Sci. Sports. 21, 496–509. 10.1111/j.1600-0838.2011.01299.x 21496106

[B197] Okada A. Miyake H. Takizawa A. Minami M. (1972). A study on the excreted catecholamines in the urine of Bobsleigh-tobogganing contestants. J. Sports Med. Phys. Fitness. 12, 71–75. 4643895

[B198] Olsen O. E. Myklebust G. Engebretsen L. Bahr R. (2004). Injury mechanisms for anterior cruciate ligament injuries in team handball: a systematic video analysis. Am. J. Sports Med. 32, 1002–1012. 10.1177/0363546503261724 15150050

[B199] Olsen S. J. Fleisig G. S. Dun S. Loftice J. Andrews J. R. (2006). Risk factors for shoulder and elbow injuries in adolescent baseball pitchers. Am. J. Sports Med. 34, 905–912. 10.1177/0363546505284188 16452269

[B200] Oppliger R. A. Case H. S. Horswill C. A. Landry G. L. Shelter A. C. (1996). American College of Sports Medicine position stand. Weight loss in wrestlers. Med. Sci. Sports Exerc. 28, ix–xii. 10.1097/00005768-199610000-00049 8926865

[B201] Otto M. Holthusen S. Bahn E. Sohnchen N. Wiltfang J. Geese R. . (2000). Boxing and running lead to a rise in serum levels of S-100B protein. Int. J. Sports Med. 21, 551–555. 10.1055/s-2000-8480 11156273

[B202] Paix B. R. (1999). Rider injury rates and emergency medical services at equestrian events. Br. J. Sports Med. 33, 46–48. 10.1136/bjsm.33.1.46 10027058 PMC1756135

[B203] Pearson S. J. Young A. Macaluso A. Devito G. Nimmo M. A. Cobbold M. . (2002). Muscle function in elite master weightlifters. Med. Sci. Sports Exerc. 34, 1199–1206. 10.1097/00005768-200207000-00023 12131263

[B204] Perkins-Ceccato N. Passmore S. R. Lee T. D. (2003). Effects of focus of attention depend on golfers' skill. J. Sports Sci. 21, 593–600. 10.1080/0264041031000101980 12875310

[B205] Perrin P. Deviterne D. Hugel F. Perrot C. (2002). Judo, better than dance, develops sensorimotor adaptabilities involved in balance control. Gait Posture 15, 187–194. 10.1016/S0966-6362(01)00149-7 11869913

[B206] Philippon M. J. Weiss D. R. Kuppersmith D. A. Briggs K. K. Hay C. J. (2010). Arthroscopic labral repair and treatment of femoroacetabular impingement in professional hockey players. Am. J. Sports Med. 38, 99–104. 10.1177/0363546509346393 19966097

[B207] Phomsoupha M. Laffaye G. (2015). The science of badminton: game characteristics, anthropometry, physiology, visual fitness and biomechanics. Sports Med. 45, 473–495. 10.1007/s40279-014-0287-2 25549780

[B208] Pino E. C. Colville M. R. (1989). Snowboard injuries. Am. J. Sports Med. 17, 778–781. 10.1177/036354658901700610 2624290

[B209] Platzer H. P. Raschner C. Patterson C. (2009). Performance-determining physiological factors in the luge start. J. Sports Sci. 27, 221–226. 10.1080/02640410802400799 19156559

[B210] Plews D. J. Laursen P. B. Kilding A. E. Buchheit M. (2012). Heart rate variability in elite triathletes, is variation in variability the key to effective training? A case comparison. Eur. J. Appl. Physiol. 112, 3729–3741. 10.1007/s00421-012-2354-4 22367011

[B211] Plisky P. J. Rauh M. J. Kaminski T. W. Underwood F. B. (2006). Star Excursion Balance Test as a predictor of lower extremity injury in high school basketball players. J. Orthop. Sports Phys. Ther. 36, 911–919. 10.2519/jospt.2006.2244 17193868

[B212] Pojskic H. McGawley K. Gustafsson A. Behm D. G. (2020). The reliability and validity of a novel sport-specific balance test to differentiate performance levels in elite curling players. J. Sports Sci. Med. 19, 337–346. 32390727 PMC7196740

[B213] Pujol N. Blanchi M. P. Chambat P. (2007). The incidence of anterior cruciate ligament injuries among competitive Alpine skiers: a 25-year investigation. Am. J. Sports Med. 35, 1070–1074. 10.1177/0363546507301083 17468379

[B214] Pyne D. B. (2021). Growing the international reach and accessibility of IJSPP. Int. J. Sports Physiol. Perform. 21, 1–2. 10.1123/ijspp.2021-0244 34157678

[B215] Raab M. Masters R. S. Maxwell J. P. (2005). Improving the 'how' and 'what' decisions of elite table tennis players. Hum. Mov. Sci. 24, 326–344. 10.1016/j.humov.2005.06.004 16081176

[B216] Reid S. A. (2003). Stress fracture of the ulna in an elite bobsled brakeman. Clin. J. Sport Med. 13, 306–308. 10.1097/00042752-200309000-00006 14501314

[B217] Reilly T. Bangsbo J. Franks A. (2000). Anthropometric and physiological predispositions for elite soccer. J. Sports Sci. 18, 669–683. 10.1080/02640410050120050 11043893

[B218] Robertson I. Arnold G. P. Wang W. Drew T. S. Nasir S. MacDonald C. . (2017). A pilot biomechanical assessment of curling deliveries: is toe sliding more likely to cause knee injury than flatfoot sliding? BMJ Open Sport Exerc Med. 3:e000221. 10.1136/bmjsem-2017-000221 29021906 PMC5633733

[B219] Rodrigues S. T. Vickers J. N. Williams A. M. (2002). Head, eye and arm coordination in table tennis. J. Sports Sci. 20, 187–200. 10.1080/026404102317284754 11999475

[B220] Roi G. S. Bianchedi D. (2008). The science of fencing: implications for performance and injury prevention. Sports Med. 38, 465–481. 10.2165/00007256-200838060-00003 18489194

[B221] Ronning R. Ronning I. Gerner T. Engebretsen L. (2001). The efficacy of wrist protectors in preventing snowboarding injuries. Am. J. Sports Med. 29, 581–585. 10.1177/03635465010290051001 11573916

[B222] Royal K. A. Farrow D. Mujika I. Halson S. L. Pyne D. Abernethy B. (2006). The effects of fatigue on decision making and shooting skill performance in water polo players. J. Sports Sci. 24, 807–815. 10.1080/02640410500188928 16815774

[B223] Rundell K. W. (1995). Treadmill roller ski test predicts biathlon roller ski race results of elite U.S. biathlon women. Med. Sci. Sports Exerc. 27, 1677–1685. 10.1249/00005768-199512000-00015 8614325

[B224] Rundell K. W. Bacharach D. W. (1995). Physiological characteristics and performance of top U.S. biathletes. Med. Sci. Sports Exerc. 27, 1302–1310. 10.1249/00005768-199509000-00010 8531629

[B225] Ryan C. G. Grant P. M. Tigbe W. W. Granat M. H. (2006). The validity and reliability of a novel activity monitor as a measure of walking. Br. J. Sports Med. 40, 779–784. 10.1136/bjsm.2006.027276 16825270 PMC2564393

[B226] Salazar W. Landers D. M. Petruzzello S. J. Han M. Crews D. J. Kubitz K. A. (1990). Hemispheric asymmetry, cardiac response, and performance in elite archers. Res. Q. Exerc. Sport. 61, 351–359. 10.1080/02701367.1990.10607499 2132894

[B227] Saltin B. Astrand P. O. (1967). Maximal oxygen uptake in athletes. J. Appl. Physiol. 23, 353–358. 10.1152/jappl.1967.23.3.353 6047957

[B228] Sandbakk O. (2018). Let's close the gap between research and practice to discover new land together! Int. J. Sports Physiol. Perform. 13:961. 10.1123/ijspp.2018-0550 30189759

[B229] Sands W. A. Smith L. S. Kivi D. M. McNeal J. R. Dorman J. C. Stone M. H. . (2005). Anthropometric and physical abilities profiles: US National Skeleton Team. Sports Biomech. 4, 197–214. 10.1080/14763140508522863 16138657

[B230] Santos J. Garcia P. (2011). A bibliometric analysis of sport economics research. Int. J. Sport Fin. 6, 222–244.

[B231] Saury J. Durand M. (1998). Practical knowledge in expert coaches: on-site study of coaching in sailing. Res. Q. Exerc. Sport. 69, 254–266. 10.1080/02701367.1998.10607692 9777662

[B232] Schmitt H. Gerner H. J. (2001). Paralysis from sport and diving accidents. Clin. J. Sport Med. 11, 17–22. 10.1097/00042752-200101000-00004 11176141

[B233] Secher N. H. (1993). Physiological and biomechanical aspects of rowing. Implications for training. Sports Med. 15, 24–42. 10.2165/00007256-199315010-00004 8426942

[B234] Seifert J. G. Luetkemeier M. J. Spencer M. K. Miller D. Burke E. R. (1997). The effects of mountain bike suspension systems on energy expenditure, physical exertion, and time trial performance during mountain bicycling. Int. J. Sports Med. 18, 197–200. 10.1055/s-2007-972619 9187974

[B235] Shanley E. Michener L. A. Ellenbecker T. S. Rauh M. J. (2012). Shoulder range of motion, pitch count, and injuries among interscholastic female softball pitchers: a descriptive study. Int. J. Sports Phys. Ther. 7, 548–557. 23091788 PMC3474308

[B236] Sherar L. B. Baxter-Jones A. D. Faulkner R. A. Russell K. W. (2007). Do physical maturity and birth date predict talent in male youth ice hockey players? J. Sports Sci. 25, 879–886. 10.1080/02640410600908001 17474041

[B237] Shilbury D. (2011). A bibliometric analysis of four sport management journals. Sport Manage. Rev. 14, 434–452. 10.1016/j.smr.2010.11.005

[B238] Sjodin B. Jacobs I. (1981). Onset of blood lactate accumulation and marathon running performance. Int. J. Sports Med. 2, 23–26. 10.1055/s-2008-1034579 7333732

[B239] Smith H. K. (1998). Applied physiology of water polo. Sports Med. 26, 317–334. 10.2165/00007256-199826050-00003 9858395

[B240] Smith M. S. Dyson R. J. Hale T. Janaway L. (2000). Development of a boxing dynamometer and its punch force discrimination efficacy. J. Sports Sci. 18, 445–450. 10.1080/02640410050074377 10902679

[B241] Soligard T. Palmer D. Steffen K. Lopes A. D. Grant M. E. Kim D. . (2019). Sports injury and illness incidence in the PyeongChang 2018 Olympic Winter Games: a prospective study of 2914 athletes from 92 countries. Br. J. Sports Med. 53, 1085–1092. 10.1136/bjsports-2018-100236 31235615

[B242] Soligard T. Steffen K. Palmer D. Alonso J. M. Bahr R. Lopes A. D. . (2017). Sports injury and illness incidence in the Rio de Janeiro 2016 Olympic Summer Games: A prospective study of 11274 athletes from 207 countries. Br. J. Sports Med. 51, 1265–1271. 10.1136/bjsports-2017-097956 28756389

[B243] Solli G. S. Tonnessen E. Sandbakk O. (2017). The Training characteristics of the world's most successful female cross-country skier. Front. Physiol. 8:1069. 10.3389/fphys.2017.01069 29326603 PMC5741652

[B244] Spencer M. Lawrence S. Rechichi C. Bishop D. Dawson B. Goodman C. (2004). Time-motion analysis of elite field hockey, with special reference to repeated-sprint activity. J. Sports Sci. 22, 843–850. 10.1080/02640410410001716715 15513278

[B245] Stapelfeldt B. Schwirtz A. Schumacher Y. O. Hillebrecht M. (2004). Workload demands in mountain bike racing. Int. J. Sports Med. 25, 294–300. 10.1055/s-2004-819937 15162249

[B246] Steen S. N. Brownell K. D. (1990). Patterns of weight loss and regain in wrestlers: has the tradition changed? Med. Sci. Sports Exerc. 22, 762–768. 10.1249/00005768-199012000-00005 2287253

[B247] Stokes K. A. Locke D. Roberts S. Henderson L. Tucker R. Ryan D. . (2021). Does reducing the height of the tackle through law change in elite men's rugby union (The Championship, England) reduce the incidence of concussion? A controlled study in 126 games. Br. J. Sports Med. 55, 220–225. 10.1136/bjsports-2019-101557 31857335

[B248] Stolen T. Chamari K. Castagna C. Wisloff U. (2005). Physiology of soccer: an update. Sports Med. 35, 501–536. 10.2165/00007256-200535060-00004 15974635

[B249] Stone R. C. Rakhamilova Z. Gage W. H. Baker J. (2018). Curling for confidence: psychophysical benefits of curling for older adults. J. Aging Phys. Act. 26, 267–275. 10.1123/japa.2016-0279 28952847

[B250] Suarez-Arrones L. Arenas C. Lopez G. Requena B. Terrill O. Mendez-Villanueva A. (2014). Positional differences in match running performance and physical collisions in men rugby sevens. Int. J. Sports Physiol. Perform. 9, 316–323. 10.1123/ijspp.2013-0069 23881362

[B251] Suarez-Arrones L. Calvo-Lluch A. Portillo J. Sanchez F. Mendez-Villanueva A. (2013). Running demands and heart rate response in rugby sevens referees. J. Strength Cond. Res. 27, 1618–1622. 10.1519/JSC.0b013e3182712755 22990568

[B252] Taddei F. Bultrini A. Spinelli D. Di Russo F. (2012). Neural correlates of attentional and executive processing in middle-age fencers. Med. Sci. Sports Exerc. 44, 1057–1066. 10.1249/MSS.0b013e31824529c2 22157879

[B253] Takahashi I. Umeda T. Mashiko T. Chinda D. Oyama T. Sugawara K. . (2007). Effects of rugby sevens matches on human neutrophil-related non-specific immunity. Br. J. Sports Med. 41, 13–18. 10.1136/bjsm.2006.027888 17035481 PMC2465143

[B254] Tarazi F. Dvorak M. F. Wing P. C. (1999). Spinal injuries in skiers and snowboarders. Am. J. Sports Med. 27, 177–180. 10.1177/03635465990270021101 10102098

[B255] Thomas J. Walker T. W. Miller S. Cobb A. Thomas S. J. (2016). The Olympic legacy: Journal metrics in sports medicine and dentistry. J. Int. Soc. Prevent. Commun. Dentistry 6, 501–508. 10.4103/2231-0762.195513 28032040 PMC5184382

[B256] Tricoli V. Lamas L. Carnevale R. Ugrinowitsch C. (2005). Short-term effects on lower-body functional power development: weightlifting vs. vertical jump training programs. J. Strength Cond. Res. 19, 433–437. 10.1519/00124278-200505000-00032 15903387

[B257] Tsekouras Y. E. Kavouras S. A. Campagna A. Kotsis Y. P. Syntosi S. S. Papazoglou K. . (2005). The anthropometrical and physiological characteristics of elite water polo players. Eur. J. Appl. Physiol. 95, 35–41. 10.1007/s00421-005-1388-2 15976998

[B258] Tyler T. F. Nicholas S. J. Campbell R. J. McHugh M. P. (2001). The association of hip strength and flexibility with the incidence of adductor muscle strains in professional ice hockey players. Am. J. Sports Med. 29, 124–128. 10.1177/03635465010290020301 11292035

[B259] Vad V. B. Bhat A. L. Basrai D. Gebeh A. Aspergren D. D. Andrews J. R. (2004). Low back pain in professional golfers: the role of associated hip and low back range-of-motion deficits. Am. J. Sports Med. 32, 494–497. 10.1177/0363546503261729 14977679

[B260] van Gent R. N. Siem D. van Middelkoop M. van Os A. G. Bierma-Zeinstra S. M. Koes B. W. (2007). Incidence and determinants of lower extremity running injuries in long distance runners: a systematic review. Br. J. Sports Med. 41, 469–480; discussion 80. 10.1136/bjsm.2006.033548 17473005 PMC2465455

[B261] van Ingen Schenau G. J. (1982). The influence of air friction in speed skating. J. Biomech. 15, 449–458. 10.1016/0021-9290(82)90081-1 7118959

[B262] van Ingen Schenau G. J. de Koning J. J. de Groot G. (1994). Optimisation of sprinting performance in running, cycling and speed skating. Sports Med. 17, 259–275. 10.2165/00007256-199417040-00006 8009139

[B263] Verhagen E. A. Van der Beek A. J. Bouter L. M. Bahr R. M. Van Mechelen W. (2004). A one season prospective cohort study of volleyball injuries. Br. J. Sports Med. 38, 477–481. 10.1136/bjsm.2003.005785 15273190 PMC1724865

[B264] Vickers J. N. Williams A. M. (2007). Performing under pressure: the effects of physiological arousal, cognitive anxiety, and gaze control in biathlon. J. Mot. Behav. 39, 381–394. 10.3200/JMBR.39.5.381-394 17827115

[B265] Vogiatzis I. Spurway N. C. Wilson J. Boreham C. (1995). Assessment of aerobic and anaerobic demands of dinghy sailing at different wind velocities. J. Sports Med. Phys. Fitness. 35, 103–107. 7500623

[B266] Volianitis S. McConnell A. K. Koutedakis Y. McNaughton L. Backx K. Jones D. A. (2001). Inspiratory muscle training improves rowing performance. Med. Sci. Sports Exerc. 33, 803–809. 10.1097/00005768-200105000-00020 11323552

[B267] Walilko T. J. Viano D. C. Bir C. A. (2005). Biomechanics of the head for Olympic boxer punches to the face. Br. J. Sports Med. 39, 710–719. 10.1136/bjsm.2004.014126 16183766 PMC1725037

[B268] Watts P. B. (2004). Physiology of difficult rock climbing. Eur. J. Appl. Physiol. 91, 361–372. 10.1007/s00421-003-1036-7 14985990

[B269] Watts P. B. Martin D. T. Durtschi S. (1993). Anthropometric profiles of elite male and female competitive sport rock climbers. J. Sports Sci. 11, 113–117. 10.1080/02640419308729974 8497013

[B270] Webster S. Rutt R. Weltman A. (1990). Physiological effects of a weight loss regimen practiced by college wrestlers. Med. Sci. Sports Exerc. 22, 229–234. 2355820

[B271] Werner S. L. Jones D. G. Guido J. A. Brunet M. E. (2006). Kinematics and kinetics of elite windmill softball pitching. Am. J. Sports Med. 34, 597–603. 10.1177/0363546505281796 16282576

[B272] Williams L. R. Walmsley A. (2000). Response timing and muscular coordination in fencing: a comparison of elite and novice fencers. J. Sci. Med. Sport 3, 460–475. 10.1016/S1440-2440(00)80011-0 11235010

[B273] Williamson I. J. Goodman D. (2006). Converging evidence for the under-reporting of concussions in youth ice hockey. Br. J. Sports Med. 40, 128–132. 10.1136/bjsm.2005.021832 16431999 PMC2492052

[B274] Wong del P. Tan E. C. Chaouachi A. Carling C. Castagna C. Bloomfield J. . (2010). Using squat testing to predict training loads for lower-body exercises in elite karate athletes. J. Strength Cond. Res. 24, 3075–3080. 10.1519/JSC.0b013e3181d65071 20838250

[B275] Wulf G. Lauterbach B. Toole T. (1999). The learning advantages of an external focus of attention in golf. Res. Q. Exerc. Sport. 70, 120–126. 10.1080/02701367.1999.10608029 10380243

[B276] Wulf G. Su J. (2007). An external focus of attention enhances golf shot accuracy in beginners and experts. Res. Q. Exerc. Sport. 78, 384–389. 10.1080/02701367.2007.10599436 17941543

[B277] Young W. McLean B. Ardagna J. (1995). Relationship between strength qualities and sprinting performance. J. Sports Med. Phys. Fitness. 35, 13–19. 7474987

[B278] Zalavras C. Nikolopoulou G. Essin D. Manjra N. Zionts L. E. (2005). Pediatric fractures during skateboarding, roller skating, and scooter riding. Am. J. Sports Med. 33, 568–573. 10.1177/0363546504269256 15722288

[B279] Zanoletti C. La Torre A. Merati G. Rampinini E. Impellizzeri F. M. (2006). Relationship between push phase and final race time in skeleton performance. J. Strength Cond. Res. 20, 579–583. 10.1519/00124278-200608000-00019 16937971

